# DNA sequence influences hexasome orientation to regulate DNA accessibility

**DOI:** 10.1093/nar/gkz283

**Published:** 2019-04-24

**Authors:** Matthew Brehove, Elan Shatoff, Benjamin T Donovan, Caroline M Jipa, Ralf Bundschuh, Michael G Poirier

**Affiliations:** 1 Department of Physics, Ohio State University, Columbus, OH 43210, USA; 2 Biophysics Graduate Program, Ohio State University, Columbus, OH 43210, USA; 3 Department of Chemistry and Biochemistry, Ohio State University, Columbus, OH 43210, USA; 4 Division of Hematology, Ohio State University, Columbus, OH 43210, USA; 5 Center for RNA Biology, Ohio State University, Columbus, OH 43210, USA; 6 Ohio State Biochemistry Program, Ohio State University, Columbus, OH 43210, USA

## Abstract

Nucleosomes, the fundamental organizing units of eukaryotic genomes, contain ∼146 base pairs of DNA wrapped around a histone H3–H4 tetramer and two histone H2A–H2B dimers. Converting nucleosomes into hexasomes by removal of a H2A–H2B dimer is an important regulatory event, but its regulation and functional consequences are not well-understood. To investigate the influence of hexasomes on DNA accessibility, we used the property of the Widom-601 Nucleosome Positioning Sequence (NPS) to form homogeneously oriented hexasomes *in vitro*. We find that DNA accessibility to transcription factors (TF) on the hexasome H2A–H2B distal side is identical to naked DNA, while the accessibility on the H2A–H2B proximal side is reduced by 2-fold, which is due to a 2-fold reduction in hexasome unwrapping probability. We then determined that a 23 bp region of the Widom-601 NPS is responsible for forming homogeneously oriented hexasomes. Analysis of published ChIP-exo data of hexasome containing genes identified two DNA sequence motifs that correlate with hexasome orientation *in vivo*, while ExoIII mapping studies of these sequences revealed they generate homogeneously oriented hexasomes *in vitro*. These results indicate that hexasome orientation, which is influenced by the underlying DNA sequence *in vivo*, is important for modulating DNA accessibility to regulate transcription.

## INTRODUCTION

All eukaryotic genomes are organized into chromatin by repeated wrapping of ∼146 base pairs of DNA around a protein octamer composed of two copies each of histones H2A, H2B, H3 and H4 (1). The structure of both chromatin and nucleosomes function to compact and control access to genomic DNA (2–4). The histone octamer is composed of three subunits: one H3–H4 tetramer and two H2A–H2B dimers (5,6), where nucleosomes are assembled both *in vivo* and *in vitro* by first the deposition of the H3–H4 tetramer and then the H2A–H2B dimers (7,8). The disassembly occurs in reverse where H2A–H2B dimers dissociate first, followed by H3–H4 tetramer dissociation (9). The step wise process of nucleosome assembly and disassembly implies that there are intermediate sub-nucleosomal complexes. Two of these nucleosome intermediates are the hexasome, which is missing one H2A–H2B dimer, and the tetrasome, which is missing both H2A–H2B dimers.

Previous biophysical studies have demonstrated by mass spectrometry that hexasomes can be reconstituted *in vitro* by salt dialysis (10). Digestion studies have shown that hexasomes protect ∼30 fewer bases than nucleosomes and data from small angle X-ray scattering experiments are consistent with about 30 bases being unwrapped on one side (11). Recently, molecular dynamics simulations of nucleosomes with one dimer removed show 40 bases unwrapped with the excess DNA pointing away from the octamer (11). Interestingly, a hexasome can be formed adjacent to a nucleosome to make a stable complex *in vitro* (12). The histone octamer and histone tetramer in this structure do not deviate significantly from the histone structure within the nucleosome (13). The combination of these studies indicate that hexasomes are unwrapped on one side by 30–40 bases and that the structure of the remaining histone core is not significantly altered by the removal of an H2A–H2B dimer.

Hexasomes can be formed by a number of mechanisms in addition to their formation as an intermediate of nucleosome assembly. Transcription through a nucleosome by RNA Pol II often induces the dissociation of an H2A–H2B dimer (14,15). This is likely involved in the rapid H2A–H2B dimer exchange within actively transcribed genes (16,17). Histone chaperones and chromatin remodeling complexes also are implicated in hexasome formation. The histone chaperones Nap1 (18,19) and FACT are reported to help remove H2A–H2B dimers (20), while FACT also facilitates H2A–H2B dimer exchange with H2A.X-H2A dimers (21). The chromatin remodeling complexes SWI/SNF and RSC slide adjacent nucleosomes into each other, resulting in the dissociation of an H2A–H2B heterodimer (22). The Swr1 remodeler exchanges H2A–H2B dimers with H2A.Z-H2B dimers (23), while INO80 may do the reverse (24). Furthermore, hexasomes significantly impact the remodeling activity of CHD1 (25). Recently, Rhee *et al.* provided evidence for the persistent presence of hexasomes near transcription start sites using ChIP-exo to determine the correlation between dimer occupancy on either side of the +1/+2 nucleosomes (26). These and other studies provide strong evidence for the formation of hexasomes *in vivo*. It is therefore important to determine their physical properties to understand how hexasomes function *in vivo*.

Nucleosomes are dynamic structures where thermal fluctuations cause nucleosomal DNA to continually unwrap and rewrap (27,28). This site exposure provides DNA binding complexes such as TF transient access to the nucleosomal DNA most predominantly in the DNA entry-exit region. Nucleosomal DNA accessibility is regulated by numerous factors including DNA sequence (29), post translational modifications (PTM) (30–32), H1 occupancy (33), and histone PTM readers (34). Furthermore, transient unwrapping on one side may influence whether nucleosomal DNA on the opposite side of the nucleosome is unwrapped as well (35). In contrast to nucleosomes, little is known about the structural dynamics of hexasomes and how the missing dimer impacts TF accessibility.

Here, we report studies on how the conversion of nucleosomes to hexasomes influences TF occupancy and TF binding/dissociation kinetics as well as how DNA sequence influences hexasome orientation. We took advantage of the recently reported observation that the Widom-601 nucleosome positioning sequence (601 NPS) binds H2A–H2B dimers asymmetrically so that H2A–H2B binds homogeneously to the left side of the 601 NPS (25). We used this property to prepare hexasomes that are homogeneously oriented such that the H2A–H2B dimer is either proximal or distal to a TF binding site. Using ensemble and single molecule fluorescence measurements we find that the TF occupancy on the H2A–H2B distal side is essentially identical to fully exposed DNA, while the TF occupancy on the proximal side of the hexasome is 2-fold lower relative to nucleosomes. This reduction is due to a 2-fold decrease in the TF binding rate, which suggests that the loss of the H2A–H2B dimer reduces the probability of DNA unwrapping. We then investigated the influence of the 601 NPS DNA sequence on hexasome orientation. As part of these DNA accessibility measurements, we inserted a TF binding site that extended 27 bp into the 601 NPS without altering its H2A–H2B asymmetric binding suggesting only a portion of the 601 sequence is important of hexasome orientation. We investigated this further by preparing 601 NPS chimeras and found that a 23 bp length of the 601 DNA sequence is fully responsible for this asymmetric H2A–H2B dimer binding. We then analyzed published ChIP-exo data of hexasome containing genes (26) and identified two 20–30 bp DNA sequence motifs that correlate with hexasome orientation *in vivo*. Analysis of sequences based on these motifs revealed they generate homogeneously oriented hexasomes *in vitro*. Together, these results show that conversion of nucleosomes to hexasomes has a dramatic impact on DNA accessibility, while the orientation of the hexasome determines which side of a nucleosome increases or decreases in accessibility. Furthermore, we provide evidence that orientation could be influenced by specific DNA sequence motifs within the genome. Overall these results support the idea that regulation of hexasome formation and orientation could be a significant regulator of DNA accessibility and ultimately transcription.

## MATERIALS AND METHODS

### Preparation of labeled DNA

All dsDNA molecules used were prepared by PCR from a plasmid that contained the nucleosome positioning sequence with either the Gal4 or LexA target sequence inserted on either the right or left side of the NPS. A list of the DNA sequences used in the study are provided in Supplemental Material (Supplementary Tables S1 and S2). The LexA binding sequence used was TACTGTATGAGCATACAGTA and the 2C Gal4 binding sequence used was CCGGAGGGCTGCCCTCCGG. To prepare the 601 chimeras and the 601 sequences with either the weak or strong dimer binding motifs, the 601 base pairs were changed using site-directed mutagenesis (Qiagen 200514). The modified 601 sequences each contained the same Gal4 site found in 601-Gal4-S, though this was not used in the mapping experiments. The oligonucleotides (Sigma Aldrich) used as primers in each PCR are listed in the Supplemental Material (Supplementary Table S3). The DNA primers used to prepare DNA for the ExoIII mapping were purchased with the 5′ end labeled with either Cy3 or Cy5. The forward DNA primer used to prepare 601-Gal4-S, 601-Gal4-W, 601-LexA-W and DNA-LexA contained a 5′ amine that was labeled with Cy3-NHS (GE Healthcare). The reverse DNA primer for preparing DNA-LexA contained an internal amine attached to the base of a dT, that was labeled with Cy5-NHS (GE Healthcare). Each fluorophore labeled DNA primer was then purified by reverse phase HPLC on a 218TP C18 column (Grace/Vydac). Following PCR, each dsDNA sample was phenol-extracted and then purified by FPLC on an UnoQ (Biorad) ion exchange column.

### Preparation of fluorophore labeled histone heterodimer, tetramer and octamer

All human histones were expressed individually in BL21-PLysS cells and purified as described previously (36). H2A–H2B dimer, H3–H4 tetramer and H2A–H2B-H3–H4 octamer were refolded as described previously (36). Briefly, lyophilized histones were resuspended in unfolding buffer (7 M guanidine-HCl, 10 mM Tris–HCl pH 7.5, 10 mM Dithiothreitol (DTT)) to <5 mg/ml and allowed to unfold for 1 h. They were then combined at equimolar ratios and dialyzed into refolding buffer (2 M NaCl, 10 mM Tris–HCl pH 7.5, 1 mM ethylenediaminetetraacetic acid (EDTA) pH8, 2 mM 2-mercaptoethanol (BME)). Full octamer was refolded with all four histones for nucleosome reconstitutions, while H2A–H2B dimer and H3–H4 tetramer were refolded separately for hexasome reconstitutions. H2AK119C and H3V35C were included in the dimer and tetramer refoldings, respectively, if they were to be labeled with Cy5. Octamer included one of these mutations for Cy5 labeling. Histone H3 always contained the C110A mutation to avoid Cy5 labeling.

Following the refolding, the cysteine mutations were labeled with Cy5 maleimide as described previously (37). Briefly, tris(2-carboxyethyl)phosphine (TCEP) was added at 10 mM to refolded histone octamer and incubated for 30 min on ice. TCEP was then removed by dialysis into 5 mM PIPES, pH 6.1, with 2 M NaCl. The sample was purged of oxygen under argon gas for 15 min. HEPES, pH 7.1 was then added to the sample to 100 mM final concentration. Cy5 maleimide was resuspended in anhydrous dimethylformamide and then added to 5-fold molar excess. The labeling reaction was incubated for 1 h at room temperature and then overnight at 4°C. The reaction was then quenched by adding DTT to 10 mM. The dimer, tetramer or octamer was then purified by gel filtration chromatography using a Superdex 200 column (GE Healthcare), which also removed the free Cy5 dye.

### Hexasome and nucleosome reconstitutions

**Figure 1.**
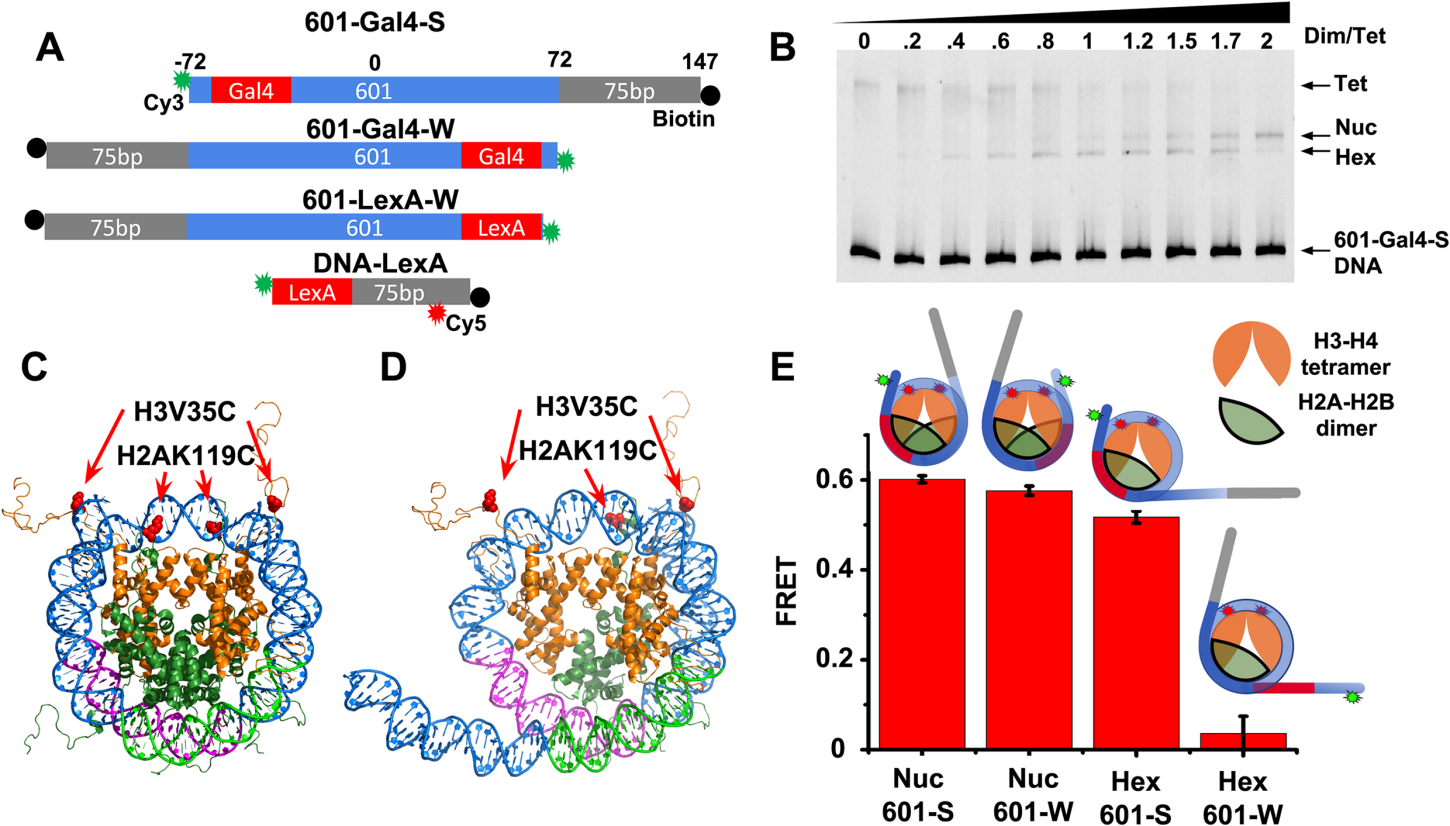
(**A**) Diagrams of the DNA constructs used in fluorescent experiments. All constructs are 5′ end-labeled with Cy3 fluorophores (green) and biotin (black) and include either Gal4 (19 bp) or LexA (20 bp) binding sites (red). 601-LexA-W has the 601 sequence truncated by six bases near the LexA site. The DNA-LexA construct also includes an internal Cy5 fluorophore (red). (**B**) EMSA of tetrasome, hexasome and nucleosome reconstitutions with an increasing ratio of H2A–H2B dimer to H3–H4 tetramer. The distinct electrophoretic mobilities of the DNA, tetrasomes, hexasomes and nucleosomes are indicated with an arrow. Each lane is labeled with the molar ratio of dimer to tetramer. A ratio of 1 to 1 maximizes the reconstitution of hexasomes. (**C**) Nucleosome crystal structure (1KX5) and (**D**) a modified nucleosome structure that represents the hexasome. One H2A–H2B dimer was removed and the region of DNA that interacts with the removed dimer was straightened using pymol. In both (C) and (D) the Cy5 labels are located at H3V35C and H2AK119C (red), and the magenta and green portions of the DNA indicate the 23 base pairs of the 601 NPS that strongly and weakly bind the H2A–H2B dimer, respectively. (**E**) Bar graph showing the FRET efficiencies of nucleosomes containing 601-Gal4-S, nucleosomes containing 601-Gal4-W, hexasomes containing 601-Gal4-S and hexasomes containing 601-Gal4-W. The diagrams above each bar are schematics of the samples being measured. The histone octamers and hexamers are in green, DNA is in blue, the Gal4 binding site is in red, and the Cy3 and Cy5 labels are in green and red stars, respectively.

Nucleosomes were prepared as described previously (36). Briefly, 50–100 pmol of octamer was combined with 20% molar excess of DNA in high salt buffer (5 mM Tris pH 8, 0.5 mM EDTA pH 8, 1 mM benzamidine, 2 M NaCl). The sample was then reconstituted by double dialysis (38) against low salt buffer (5 mM Tris pH 8, 0.5 mM EDTA and 1 mM benzamidine). Hexasomes were reconstituted identically except that dimer was varied from no dimer to a dimer:tetramer ratio of 2:1. The optimal ratio was determined to be 1:1 as expected (Figure 1B). Reconstituted nucleosomes and hexasomes were then purified by sucrose gradient centrifugation for 22 h at 41k rpm, 4°C in a Beckman-Coulter Ti-41 swinging bucket rotor using a 5–30% gradient. Gradient fractions were analyzed by Electromobility Shift Assays (EMSA) with native Polyacrylamide Gel Electrophoresis (PAGE). Fractions containing the desired hexasomes or nucleosomes were pooled, concentrated and then stored on ice. Final purified samples were then assessed by EMSA (Supplementary Figures S1–S3).

### Preparation of Gal4

The Gal4 expression vector was prepared by cloning residues 1–147 of the Gal4 gene from *Saccharomyces cerevisiae* into a pET28a vector between the NdeI and BamHI sites. This added a 6-His tag to the N-terminus. Gal4 was expressed in *Escherichia coli* BL21(DE3)pLysS cells (Invitrogen) by inducing with 0.2 mM isopropyl β-d-1-thiogalactopyranoside (IPTG) for 3 h in growth media containing 100 mM zinc acetate (ZnAc). Cells were pelleted and resuspended in Buffer A (50 mM Tris–HCl pH 8, 200 mM NaCl, 10 mM imidizole, 10 mM BME, 20μM ZnAc, 1 mM DTT, 1 mM phenylmethylsulfonyl fluoride (PMSF)). After harvesting, 20 mg/ml each of pepstatin A and leupeptin were added to the cells, which were then lysed by sonication and centrifuged at 23,000 g for 20 min. Affinity purification was performed by binding the supernatant to Ni-NTA agarose resin in solution, then packing the resin into a reusable column and eluting with 200 mM imidizole. After collection, the sample was dialyzed into Buffer C (25 mM Tris–HCl pH 7.5, 200 mM NaCl, 20 μM ZnAc, 1 mM DTT, 1 mM PMSF). The sample was further purified by HPLC on a TSKgel^®^ SP-5PW Column (Tosoh biosciences). The sample was eluted by a gradient of 200–600 mM NaCl in 25 mM Tris–HCl pH 7.5, 1 mM DTT and 1 mM PMSF. Pooled fractions were dialyzed into 10 mM HEPES pH 7.5, 200 mM NaCl, 10% glycerol, 1 mM DTT, 10 μM ZnCl_2_, and 1 mM PMSF for flash freezing and storage.

### Preparation of LexA

LexA was expressed in *E. coli* BL21(DE3)pLysS cells (Invitrogen) by inducing with 0.2 mM IPTG for 2 h. Purification was based on published protocols (39). Cells were harvested by centrifugation and resuspended at 50 ml per 1 l starting culture in Buffer A (50 mM Tris–HCl pH 8.0, 200 mM NaCl, 1 mM DTT, 0.5 mM EDTA, 10% w/v sucrose). The cells were lysed with freeze-thaw cycles and centrifuged at 30,000 g for 30 min to pellet aggregates. Polymin P was added to the supernatant to 0.35% final concentration at 4°C to precipitate the DNA. After the removal of the DNA precipitate, LexA was precipitated by adding 0.4 g ammonium sulfate per mL of solution at 4°C. The precipitate was isolated, resuspended in Buffer A, and then re-precipitated as before. LexA was then dialyzed into Buffer B (20 mM potassium phosphate pH7, 0.1 mM EDTA, 10% glycerol, 1 mM DTT) with 500 mM NaCl. Next, the sample was purified using a HiTrap Heparin HP column (GE Healthcare) in Buffer B with a 200–800 mM NaCl gradient. Fractions that contained LexA were pooled and loaded onto a CHT Ceramic Hydroxyapatite column (BioRad) in 50 mM phosphate pH 7, 10% glycerol, 1 mM DTT, 0.5 mM CaCl_2_ with a gradient of 50–200 mM NaCl. Pooled fractions were then dialyzed into 10 mM PIPES, 0.1 mM EDTA, 10% glycerol and 200 mM NaCl before being flash frozen and stored.

### Exonuclease III mapping of nucleosome and hexasome positions

Exonuclease III (ExoIII) (NEB) digestions were performed in a heated-lid thermocycler at 37°C for 5 min. The final concentrations of ExoIII used were 0.003, 0.01, 0.03, 0.1 and 0.3 units/μl. 0.4 pmol of hexasomes or nucleosomes were added to a final concentration of 13 nM. Reactions were performed in 1× NEB Buffer 1. After incubation the reaction was quenched with equal volume formamide. Samples were denatured at 95°C for 5 min and then analyzed by denaturing PAGE (15% 29:1 acrylamide, 7 M urea, 90 mM boric acid, 90 mM Tris–HCl, 1 mM EDTA, pH 8) using 26 W for 2 h.

### Ensemble fluorescence measurements

Förster Resonance Energy Transfer (FRET) efficiencies were determined from fluorescence spectra as described previously (40). Ensemble fluorescence experiments were performed in a Fluoromax-4 (Horiba) photon-counting steady-state fluorometer at room temperature. Emission spectra were taken for both the Cy3 donor and Cy5 acceptor fluorophores. Cy3 was excited at 510 nm, and emission was measured from 550 to 750 nm. Cy5 was excited at 610 nm, and emission was measured from 650 to 750 nm. The total acceptor (Cy5) fluorescence emission (*F*) was calculated by integrating the fluorescence spectrum from 656 to 674 nm (the Cy5 emission peak) after subtracting out background fluorescence from the buffer and Cy3 emission. The FRET efficiency (*E*) was then calculated using the (ratio)_A_ method as described previously (41) with *E =* 2(*ϵ*^A^_610_*F*^A^_510_/*F*^A^_610_ –*ϵ*^A^_510_)/(*ϵ*^D^_510_*d*^+^). The superscripts refer to the donor (D) and acceptor (A) fluorophores, and the subscripts refer to the illumination wavelengths (510 nm for donor excitation and 610 nm for direct acceptor excitation). A prefactor of two reflects the presence of two acceptor molecules per donor molecule. *d^+^* is the donor labeling efficiency, which is equal to 1. *F*^A^_510_ is the fluorescence emission of the acceptor after the subtraction of overlapping donor emission when excited at 510 nm. *F*^A^_610_ is the fluorescence emission of the acceptor when excited at 610 nm. *ϵ*^A^_610_, *ϵ*^A^_510_ and *ϵ*^D^_510_ are the molar extinction coefficients of the acceptor at 510 and 610 nm and the donor at 510 nm.

Protein Induced Fluorescence Enhancement (PIFE) measurements for quantifying LexA binding to DNA and hexasomes were determined from Cy3 and Cy5 emission spectra. The Cy3 fluorophore was placed on the 5′ end of the DNA 1 base away from the LexA binding site. LexA binding to its site increases the Cy3 fluorescence by a factor of 2. Cy5 fluorescence is used to control for variations in sample concentration since Cy5 emission is not influenced by LexA binding. The Cy5 label was attached to the H2A–H2B dimer for the hexasome sample and to the LexA-DNA for the DNA only measurements. The Cy3 emission was integrated from 560 to 580 nm (*F*^D^) and the Cy5 emission was integrated from 656 to 674 nm (*F*^A^). The reported PIFE signal was calculated as *F*^A^*/F*^D^ and then normalized to 1 at zero [LexA].

### Ensemble TF binding assays

Changes in FRET efficiency and PIFE were used to quantify the accessibility of hexasomal DNA to TF (Gal4 or LexA) binding. The FRET measurements were done in a 60 μl quartz cuvette with 0.5 nM of either hexasomes or nucleosomes in a buffer containing 130 mM NaCl, 10 mM Tris–HCl pH 8, 0.01% Tween 20, 10% glycerol. The TF Gal4 was titrated from 0 to 1000 nM. The normalized change in FRET efficiency measurements were fit to a non-cooperative binding isotherm: *E* = *E*_F_ + (*E*_0_ − *E*_F_)/(1 + [TF]/*S*_1/2_) where *E* is the FRET efficiency, *S*_1/2_ is the concentration at which the FRET efficiency has decreased by half, and *E*_0_ and *E*_F_ are the minimum and maximum FRET efficiencies, respectively.

The PIFE measurements were done in a 2 ml quartz cuvette with 0.2 nM hexasomes or DNA in a buffer containing 130 mM NaCl, 10 mM Tris pH 8, 10% glycerol, 0.005% Tween 20, 0.1 mg/ml BSA. The LexA TF was titrated from 0 to 10 nM. The Cy3 PIFE binding measurements were fit to the same non-cooperative binding isotherm as the FRET measurements, where E is the Cy3 fluorescence emission, and *E*_0_ and *E*_F_ are the minimum and maximum Cy3 fluorescence emission.

### Single molecule TIRF instrumentation

The smTIRF system used has been described previously (36). The setup includes an IX71 inverted microscope (Olympus), and 532 and 638 nm diode lasers (Crystal Laser) for excitation. The excitation beams were expanded and then focused through a quartz prism (Melles Griot) at the surface of a quartz flow cell at an angle that creates total internal reflection, which minimizes background from the excitation illumination (42). The fluorescence emission from fluorophore-labeled tethered molecules was captured by a 60× water immersion objective (Olympus), split into Cy3 and Cy5 emission channels with a DualView optical assembly and imaged with a PhotonMax EMCCD camera (Princeton Instruments).

The smTIRF measurements were carried out in lab-assembled flow cells as previously described (43). Briefly, the flow cells were constructed with Quartz microscope slides (G. Finkenbeiner) functionalized with poly-ethylene glycol (PEG, Laysan Bio, MPEG-SVA-5000) and biotin–PEG (Laysan Bio, Biotin-PEG-SVA-5000). The quartz slide and a glass coverslip were assembled with a layer of patterned parafilm to make the flow cell. The quartz slides and glass coverslips were cleaned with ethanol, sonicated in toluene, and then subjected to a Piranha solution. They were silanated with 2% (v/v) 3-aminopropyl-triethoxysilane (MP biomedicals 215476680) in acetone. The quartz slides were then functionalized using a 100:1 mass ratio mixture of mono-functional PEG to biotin–PEG at 10% (w/v) PEG in 0.1 M potassium tetraborate pH 8.1.

### smFRET measurements

Each smTIRF measurement was done as previously described (36). In summary, a new quartz flow cell that was first incubated for 5 min with 1 mg/ml BSA in Buffer A (130 mM NaCl, 10 mM Tris–HCl pH 8, 0.0075% Tween 20, 10% glycerol), followed with a 5 min incubation with 20 μg/ml streptavidin in Buffer A. The biotin-functionalized sample (hexasomes or nucleosomes) in Buffer A was incubated in the flow cell for 5 min for surface attachment through the streptavidin labeled surface. Unattached sample was then washed out, the imaging buffer was added to the flow cell with a set concentration of either LexA or Gal4, and then the slide was placed onto the microscope. The imaging buffer for the smFRET experiments was Buffer A with 0.0115% cyclooctatetraene, 0.0135% nitrobenzyl alcohol, 1.6% glucose, 0.45 mg/ml glucose oxidase, 22 μg/ml catalase, 0.5 mg/ml Trolox and 40 mM Tris–HCl. To acquire the smTIRF data, the sample was first illuminated with the 638 nm laser to directly excite the Cy5 acceptor. This image gave the location of each Cy5 labeled molecule. The illumination was then switched to 532 nm to observe smFRET. Videos were taken at 5 Hz for 400 s. Three separate flow cells were prepared for each TF concentration measured to estimate uncertainty.

### smFRET data analysis

The smTIRF videos were analyzed as previously described (36). Directly excited Cy5 was used to identify the location of each molecule on the surface. For nucleosomes and hexasomes, this allowed us to verify that the molecule contained at least one H2A–H2B dimer. All traces were screened for the presence of both fluorophores, the presence of two FRET states, and for anticorrelated Cy3–Cy5 fluctuations. All traces that satisfied these criteria were then used for further analysis and are available on Zenodo at https://doi.org/10.5281/zenodo.2646530. After photobleaching events were removed, the traces were fit to a Hidden Markov Model using the software package vbFRET (44). The dwell time of each state in the calculated idealized two-state time series was tabulated for at least 700 molecules. The cumulative sum of the high and low FRET states were each fit to the integral of an exponential. The result of the fit was used to determine the binding rate (in the case of high FRET dwell times) or dissociation rate (in the case of low FRET dwell times). These rates were calculated for each of the three repeat measurements to estimate the measurement uncertainty.

### Genomic data sets

To investigate the role of DNA sequence on hexasome asymmetry *in vivo* we used ChIP-exo data that mapped histones H2A, H2B, H3 and H4 in yeast genes. Supplementary Table S2 of (26) contains dyad positions of +1, +2 and +3 nucleosomes obtained from MNase digestion and Supplementary Table S3 of (26) contains ChIP-exo tag counts of H2A, H2B, H3, and H4. Supplementary Table S4 of (26) contains TFIIB occupancy group gene expression data. All reference genome and yeast gene annotation data was obtained from the Saccharomyces Genome Database (45). All scripts, including the calculation of *P*-values, were written in Python and are available at https://github.com/bundschuhlab/PublicationScripts/tree/master/HexasomeOrientationAndAccessibility.

### Hexasome identification

Nucleosomes were defined as encompassing 74 base pairs on either side of the dyad positions defined by MNase digestion. Total H2B and H4 tag count levels upstream and downstream of the dyad positions were summed. If the total H2B or H4 tag count level upstream of the dyad was at least 2-fold larger than the total tag count level downstream of the dyad, or vice versa, the nucleosome was deemed asymmetric in that histone. If the total H2B or H4 tag count levels upstream and downstream of the dyad were within 1.3-fold of each other, the nucleosome was deemed symmetric in that histone. If a dyad position was deemed asymmetric in H2B and symmetric in H4 then it was determined to be a hexasome, either upstream or downstream biased in gene direction. If a dyad position was deemed symmetric in H2B and H4 it was determined to be a nucleosome.

### Positional nucleotide distributions

Using the reported dyad positions the genomic nucleotide (A, T, C, or G) for each distance from the dyad was obtained from the reference genome R55–1-1 (November 2006) for all upstream and downstream biased hexasomes and all nucleosomes. The frequency of each nucleotide at each position relative to the dyad was counted separately for nucleosomes and for upstream biased and downstream biased hexasomes. The frequencies were normalized to create positional nucleotide distributions, which give the probability that a nucleotide, A, T, C or G, appears at a particular position relative to the dyad. For nucleosomes in Crick strand genes, the reverse complemented nucleotides were used. Distributions from upstream and downstream biased hexasomes were divided by each other to obtain ratios of nucleotide frequencies. The same analysis was also performed for dinucleotides formed by two consecutive genomic nucleotides. *P*-values for the significance of the nucleotide frequency ratios were calculated by forming 14 groups of 10 values and then calculating a two-sided *t*-test with a null hypothesis of 1.0 for each group. Those *P*-values were corrected for multiple testing using the Bonferroni correction. We also calculated chi-squared *P*-values to determine if there were any significant correlations between orientation and position in the gene, orientation between positions in the same gene, and orientation and expression levels.

### Hexasome motif discovery

Sequences of upstream biased hexasomes, downstream biased hexasomes and nucleosomes were separately given to MEME (46) for motif discovery. Five motifs were identified for each set of sequences using the default background model. Other backgrounds were tested but none significantly improved motif significance. Motifs in hexasome sequences that were identified as similar to motifs in nucleosome sequences by TOMTOM (46,47) with a *P*-value of <10^−10^ were ignored.

### Design of artificial biasing sequences

Oriented hexasome sequences were designed by replacing either the upstream or downstream 23 bp region of the 601 sequence that was used in the *in vitro* experiment. Many potential sequences were generated by replacing one of the 23 bp regions by a sequence generated from the nucleotide frequencies as determined above. Potential sequences with replaced 23 bp regions were then scored by FIMO (48) for matches to motifs identified by MEME and two sequences with highly significant *P*-values (*P* < 10^−5^) were retained for experimental testing.

## RESULTS

### Reconstitution of homogeneously positioned and oriented hexasomes

To investigate the DNA accessibility at a specific site within hexasomes, uniformly oriented and positioned hexasomes need to be prepared. However, it has not been clear how to prepare homogeneously oriented hexasomes because of the symmetry about the nucleosome dyad. Recently, the Bowman lab reported that the 601 NPS forms hexasomes with a homogenous orientation where the H2A–H2B dimer binds almost exclusively to the left side of the 601 NPS (25). To take advantage of this 601 NPS property to study site specific DNA-protein binding, we inserted a 19 base pair Gal4 binding site into the 601 NPS between base pair positions −45 to −63, so that it extends 27 base pairs into the nucleosome (Figure 1A). The negative and positive base pair numbers indicate that the base pair is left and right of the 601 center, respectively. We hypothesized that changing the first 27 base pairs of the 601 NPS would not impact its influence on hexasome orientation. This is based on the nucleosome crystal structure (1), DNA unzipping experiments through a nucleosome containing the 601 NPS (49), and a free energy landscape analysis of these unzipping experiments (50). They all indicate that the first 25–30 base pairs of the 601 NPS are not essential for H2A–H2B binding to DNA.

To investigate if insertion of the Gal4 site into the 601 NPS altered the preferential H2A–H2B binding to the left side of the 601 sequence, we reconstituted hexasomes and nucleosomes with the 601-Gal4-S DNA (Figure 1A) and purified H3–H4 tetramer and H2A–H2B dimer that is Cy5 labeled at H2AK119C (Figure 1C-D). ‘S’ indicates the Gal4 site is on the Strong H2A–H2B binding (left) side of the 601 NPS (25). We included a 75 base pair extension on the right side of the 601 sequence and a biotin attached to the right 5′ end for single molecule Total Internal Reflection Fluorescence (smTIRF) measurements. To optimize hexasome reconstitutions, we varied the concentration of Cy5 labeled H2A–H2B dimers, while keeping the DNA and H3–H4 tetramer concentrations fixed (Figure 1B). We analyzed the reconstitutions with EMSA using native PAGE and as expected found that a ratio of one H2A–H2B dimer to one H3–H4 tetramer maximizes the formation of a band that has a mobility consistent with the formation of hexasomes (25). Interestingly, the hexasomes formed a single, well-defined band, suggesting they are located at a single position within the 601-Gal4-S sequence.

**Figure 2.**
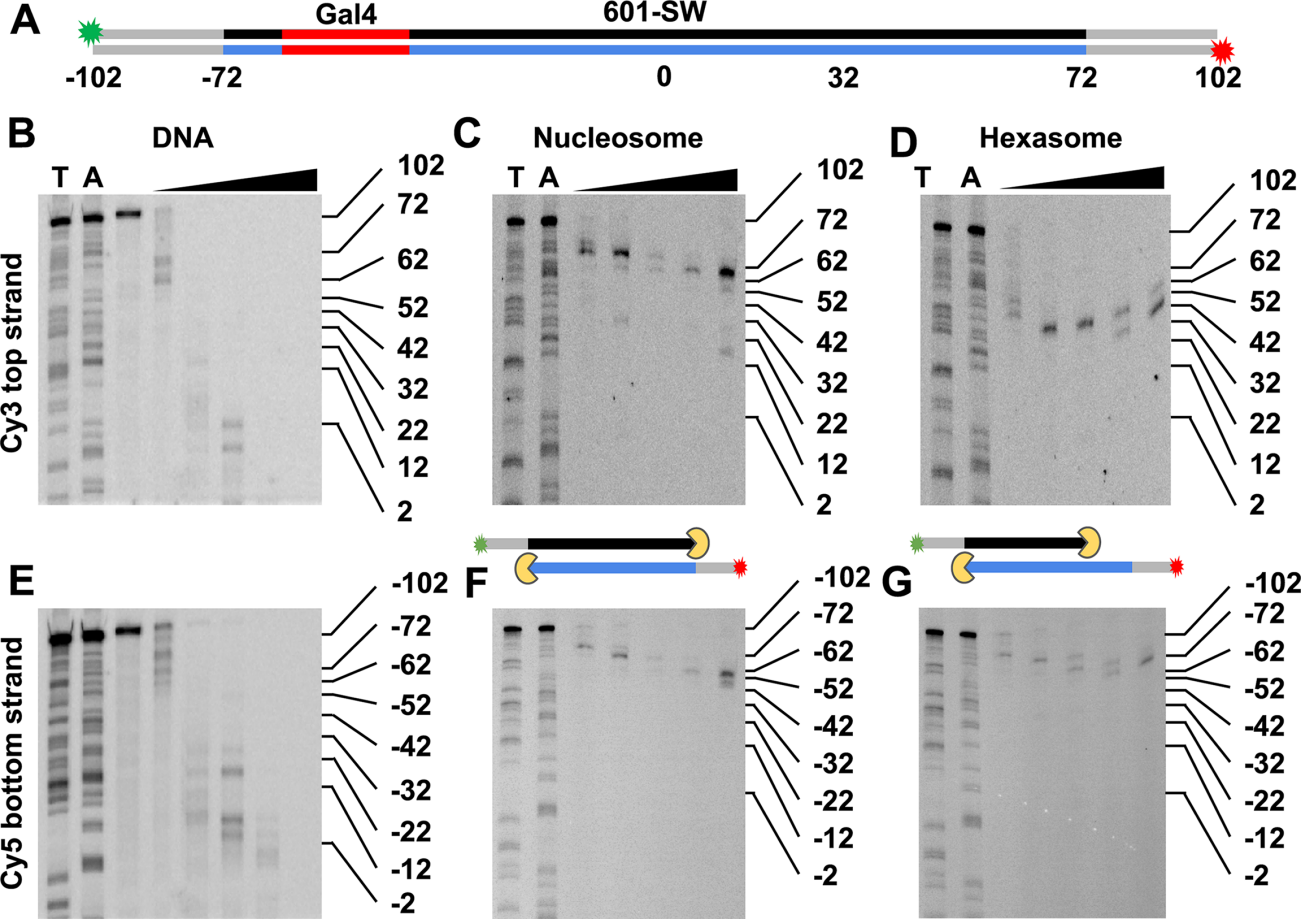
(**A**) Diagram showing both strands of the 601-SW construct for ExoIII mapping. The construct has Cy3 (green) and Cy5 (red) labels at the 5′ ends of the top and bottom strands, respectively. The DNA strands are color coded with grey representing linker DNA, the red region is the location of the Gal4 binding site (base pairs −65 to −46), the black represents the top strand and the blue represents the bottom strand the 601 nucleosome positioning sequence (NPS). (**B**–**D**) Cy3 Images of 15% denaturing PAGE of ExoIII digested 601-SW DNA, nucleosomes, and hexasomes, respectively. This visualizes the top DNA strand and indicates ExoIII stall sites on the right side of the dyad symmetry axis. Lanes T and A contain DNA sequencing ladders with ssDNA lengths terminated with a thymine or adenosine. The triangle indicates the lanes with ExoIII digested sample with 0.003, 0.01, 0.03, 0.1 and 0.3 units/μl of ExoIII for 5 minutes at 37°C. (**E**–**G**) Cy5 images of the same gels in B-D visualizing the bottom DNA strand. The 601-SW gels also include a lane with undigested DNA. The diagrams between the nucleosome and hexasome gels indicate the ExoIII (yellow) digestion stall positions. The nucleosome stall positions are at −72 and +72, while the hexasome stall positions are at −72 and +32.

We then investigated with ExoIII nucleosome mapping if integration of the Gal4 sequence into the left side of the 601 NPS influenced the preferential binding of the H2A–H2B dimer to the 601 left side. To do this, we separately prepared sucrose gradient purified hexasomes and nucleosomes with the 601-SW DNA (Figure 2A), which retained the Gal4 site and includes an additional 30 base pairs on each side of the NPS. The 601-SW DNA was Cy3 and Cy5 labeled on the left and right 5′ ends, respectively, so they could be imaged separately within the same gel. We carried out ExoIII mapping of both hexasomes and nucleosomes and then analyzed the digestions with denaturing PAGE. We find the nucleosome stalls ExoIII at the beginning of the 601 NPS on both the left and right sides (Figure 2C and F), as previously observed (51). In contrast, the hexasome stalls ExoIII on the left side identically to the nucleosome, while the right side has completely lost the stall site at the beginning of 601 NPS. Instead, there is a clear stall site ∼40 bp into the right side of the 601 NPS (Figure 2D and G), which agrees with previous reports that the hexasome protects 30–40 DNA bp less from enzymatic cleavage than nucleosomes (11,52). If the H2A–H2B dimer were to incorporate on either side of the H3–H4 tetramer, the protection patterns for both sides of the NPS would be identical. Therefore, the ExoIII mapping strongly indicates that the 601-Gal4-S NPS retains the preferential H2A–H2B dimer binding on the left side of the 601 sequence (25) and that hexasomes are homogeneously positioned and oriented.

### Hexasomes are largely wrapped on the side proximal to the H2A–H2B heterodimer, and largely unwrapped on the side distal to the H2A–H2B heterodimer

After establishing that the 601 NPS with the Gal4 binding site retains its ability to homogeneously position and orient hexasomes, we prepared Cy3-Cy5 labeled hexasomes for FRET efficiency measurements of DNA unwrapping to investigate the extent that 601 NPS is wrapped on both the H2A–H2B proximal and distal sides. Hexasomes and nucleosomes were separately reconstituted with 601-Gal4-S and 601-Gal4-W DNA constructs (Figure 1A). The 601-Gal4-W construct has the Gal4 site on the H2A–H2B distal (right) side of the 601 NPS along with the Cy3 fluorophore on the 5′ end of the 601 NPS. We reconstituted nucleosomes and hexasomes with H3–H4 tetramer that is Cy5 labeled at H3V35C and unlabeled H2A–H2B dimer, thus assuring a Cy5 acceptor on both sides of either the nucleosome or hexasome (Figure 1C and D). The Cy5 fluorophore undergoes efficient FRET with DNA attached Cy3 within nucleosomes containing either the 601-Gal4-S or the 601-Gal4-W DNA molecule (Figure 1E), as expected based on the nucleosome structure (1) and as previously reported (28). Hexasomes containing 601-Gal4-S resulted in the nearly identical FRET efficiency as compared to nucleosomes that contain either the 601-Gal4-S or the 601-Gal4-W DNA (Figure 1E). In contrast, hexasomes containing the 601-Gal4-W DNA showed no detectable FRET. This indicates that the H2A–H2B proximal DNA is wrapped similarly within hexasomes and nucleosomes, while nucleosomal DNA distal to the H2A–H2B dimer is largely unwrapped.

While the average FRET efficiency is zero on the H2A–H2B distal side of the hexasome, rare transient wrapping of the DNA could occur through DNA interactions with the H3 αN helix (53). To investigate this, we used smFRET to detect transient FRET fluctuations from 601-Gal4-W hexasomes that were tethered to a quartz slide (36). We identified 261 hexasomes that contained both Cy3 and Cy5 molecules and then measured the donor and acceptor emission during Cy3 excitation for 400 s with an acquisition rate of 200 ms. We did not detect a single high FRET fluctuation among all 261 molecules. In contrast, we identified 173 601-Gal4-S nucleosomes and 95% were continually in a high FRET state. This is consistent with the observation that nucleosomes rewrap on the ms time scale, which is too fast to detect with our 200 ms exposure time. These results imply that if the DNA on the H2A–H2B distal side of the hexasome transiently interacts with the H3 αN helix it must unwrap much faster than our 200 ms exposure time. We conclude that we find no evidence for transient DNA wrapping on the H2A–H2B distal side of the hexasome.

### DNA accessibility on the side distal to the H2A–H2B heterodimer is identical to dsDNA

Three observations indicate that the DNA near the entry-exit region of the nucleosome is largely unwrapped on the H2A–H2B distal side of the hexasome. (i) The FRET efficiency of the 601-Gal4-W hexasomes is zero (Figure 1E). (ii) The smFRET measurements of the hexasome with the Cy3-Cy5 FRET pair on the H2A–H2B distal side do not show transient DNA rewrapping. (iii) Hexasomes protect 110–120 bp of DNA from ExoIII digestion (Figure 2). However, TF binding within the 30–40 bp section of unwrapped DNA could have altered occupancy relative to duplex DNA alone. This DNA remains near the histone hexamer, so the histone tails could interact with the TF binding site and the TF itself. Also, the binding of two proteins to adjacent DNA sites impact their affinities through the DNA (54), suggesting that the DNA-histone binding could impact TF binding to a site adjacent to the histone hexamer.

To investigate this, we prepared hexasomes with 601-LexA-W DNA (Figure 1A), where the LexA binding site is inserted on the right side of the 601 sequence. We decided to use LexA for measuring site accessibility on the dimer distal side of hexasomes relative to DNA because of the time needed for TF–DNA binding to reach equilibrium. LexA-DNA binding at the LexA target site comes to equilibrium on the scale of minutes, while Gal4-DNA binding takes over an hour to reach equilibrium (55). We used PIFE to detect LexA binding to the site, since LexA binding causes a 2-fold increase in Cy3 fluorescence (43). Because PIFE is highly distance dependent, the Cy3 fluorophore was attached at the 5′ end one bp from the LexA sequence. This required the first 7 bp of the 601 sequence on the right side to be removed. This truncation is unlikely to affect binding dynamics because we found no evidence of transient wrapping with smFRET and molecular dynamics studies also showed no evidence of transient wrapping (11).

**Figure 3.**
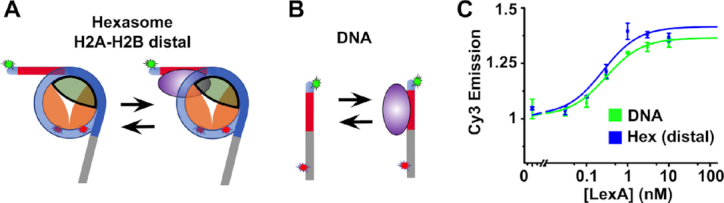
Diagrams showing a hexasome (**A**) and DNA molecule (**B**) in a bound and unbound state. The H3–H4 tetramer is in orange, the H2A–H2B dimer is in green, the DNA is in blue, the LexA site is in red, the LexA TF is in purple, and the Cy3 and Cy5 labels are green and red stars respectively. (**C**) Normalized PIFE signal vs LexA concentration for hexasomes with 601-LexA-W DNA, which has the LexA site on the H2A–H2B distal side, (blue) and naked LexA-DNA (green). The plots are fit to binding isotherms with a *S*_1/2_ of 0.25 ± 0.07 nM (hexasomes) and 0.3 ± 0.03 nM (DNA). All error bars represent standard error in the mean for three measurements.

To investigate the impact of the hexasome on TF occupancy within the H2A–H2B distal side, we carried out PIFE measurements of LexA titrations with both hexasomes (601-LexA-W, Figures 1A and 3A) and duplex DNA (DNA-LexA, Figures 1A, 3B). We find that the change in PIFE fits to a noncooperative binding isotherm with an *S*_1/2_ of 0.25 ± 0.07 nM and 0.3 ± 0.03 nM for hexasomes and DNA, respectively (Figure 3C). The *S*_1/2_ is the concentration of Gal4 required to bind 50% of either hexasomes or DNA. PIFE is strongly system dependent, so it is important to confirm that PIFE is an accurate measure of binding. We previously used EMSAs to detect LexA binding to DNA and the *S*_1/2_ determined by EMSA is similar to the *S*_1/2_ determined by PIFE (55). Therefore, these results indicate that the DNA that would be wrapped into the nucleosome but is exposed by the hexasome has an accessibility that is nearly identical to that of DNA alone.

### DNA accessibility on the side proximal to the H2A–H2B heterodimer is 2-fold lower than in nucleosomes

**Figure 4.**
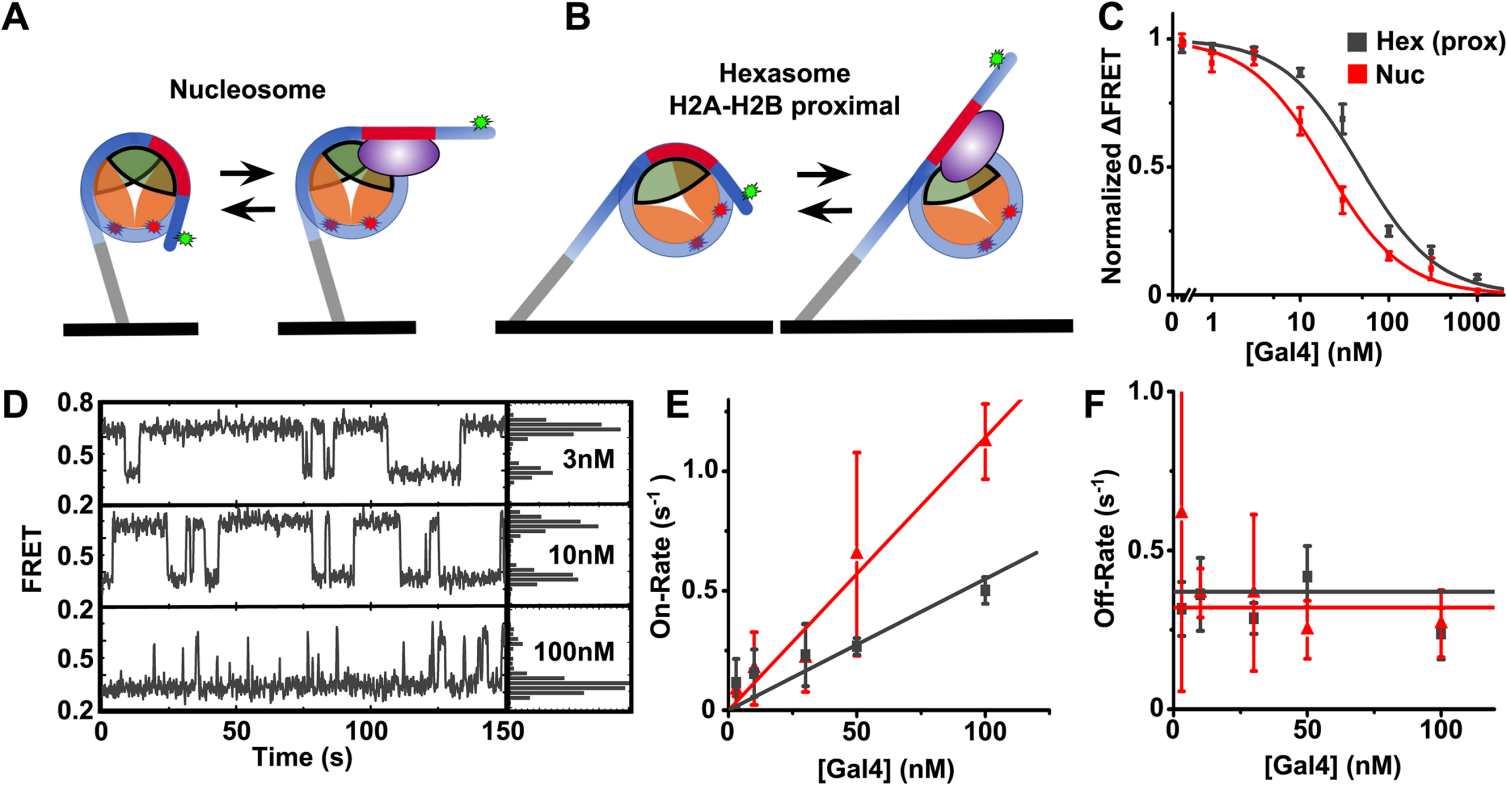
Diagrams showing a nucleosome (**A**) and hexasome (**B**) tethered to the slide surface in a bound and unbound state. The quartz slide surface is in black, the H3–H4 tetramer is in orange, the H2A–H2B dimer is in green, the DNA is in blue, the Gal4 site is in red, the Gal4 TF is in purple, and the Cy3 and Cy5 labels are green and red stars respectively. Both the heasomes and nucleosomes contained the 601-Gal4-S DNA. (**C**) Normalized ensemble FRET efficiency vs Gal4 concentration for both hexasomes with the Gal4 site on the H2A–H2B proximal side (grey) and nucleosomes (red). The plots are fit to binding isotherms with a *S*_1/2_ of 47 ± 4 nM (hexasomes) and 19.6 ± 0.3 nM (nucleosomes). (**D**) Example FRET versus time traces for hexasomes at 3, 10 and 100 nM Gal4. The histograms to the right show relative occupancy of each FRET state in the trace shown. (**E**) Gal4 on-rate to hexasomes (gray) and nucleosomes (red) for increasing [Gal4]. Each plot was fit to a linear function with a binding rate of 0.011 ± 0.004 s^−1^ nM^−1^ (nucleosomes) and 0.0052 ± 0.0005 s^−1^ nM^−1^ (hexasomes). (**F**) Gal4 off-rate from hexasomes and nucleosomes for increasing [Gal4]. Both plots were fit to a horizontal line with an unbinding rate of 0.37 ± 0.05 s^−1^ (hexasomes) and 0.32 ± 0.03 s^−1^ (nucleosomes). All errors bars represent standard error in the mean for three measurements.

Nucleosomal DNA spontaneously unwraps, allowing transcription factors (TF) such as Gal4 to bind to their target sites within the nucleosome (28,55). Our FRET efficiency measurements indicate that the DNA on the H2A–H2B proximal side of the hexasome is wrapped qualitatively similarly to DNA within nucleosomes. However, the absence of an entire H2A–H2B dimer could quantitatively impact DNA accessibility. In order to determine DNA accessibility on the H2A–H2B proximal side of the hexasome, we prepared sucrose gradient purified hexasomes and nucleosomes with the 601-Gal4-S DNA and Cy5 labeled H2AK119C (Figures 1A, 4A-B). Within these fully wrapped hexasomes and nucleosomes, the Cy3 donor efficiently undergoes energy transfer with the Cy5 acceptor. We then detected Gal4 occupancy at its target sequence since Gal4 binding traps the hexasome or nucleosome in a partially unwrapped state with low FRET efficiency. We decided to use Gal4 instead of LexA, based on the residence time of the TF at its site within nucleosomes. The residence time of LexA at its site within nucleosomes is about 0.3 s, which is close to the limit of our smTIRF detection since we acquire at 5 Hz and would only allow us to detect slower changes in dissociation rates. In contrast, the Gal4 residence time at the 2C binding site is ∼3 s (see below), which allows for detection of either an increase or decrease in the dissociation rate.

To compare the accessibility of Gal4 binding to its site in hexasomes relative to nucleosomes we first determined the Gal4 S_1/2_ for binding both hexasomes and nucleosomes. We determine the S_1/2_ from titrating Gal4 with constant hexasome or nucleosome concentration and measure the normalized Gal4-induced change in FRET efficiency (Figure 4C), which fits to non-cooperative binding isotherms (see Methods for details). We find that the *S*_1/2_ for Gal4 binding to hexasomes is 47 ± 4 nM, while Gal4 binding to nucleosomes is 19.6 ± 0.3 nM (Figure 4C). This implies that the Gal4 occupancy within the H2A–H2B proximal side of the hexasome is reduced by a factor of 2.4 ± 0.2, suggesting nucleosome accessibility is reduced by this amount. Interestingly, this reduction in accessibility is similar to that induced by single histone post translational modifications within the nucleosome entry-exit region, including H3K56 acetylation (32,37) and H3Y41 phosphorylation (40).

The characteristic concentration, *S*_1/2_, for Gal4 to bind within the nucleosome depends on both the Gal4 binding and dissociation rates (55). The binding rate is influenced by the probability that the site is partially unwrapped and exposed, while the Gal4 dissociation rate depends on other intrinsic properties of Gal4 and the nucleosome. Since both rates are dramatically influenced by the nucleosome (55), the 2-fold difference in Gal4 occupancy within hexasomes and nucleosomes could be due to changes in Gal4 binding and/or dissociation rates. To investigate this, we carried out smFRET measurements (Figure 4A and B), which separately detect the dwell times of the wrapped/unbound and unwrapped/bound states of individual hexasomes and nucleosomes (Figure 4D). We fit the cumulative sum of the dwell times of each state to determine the binding and dissociation rates (Supplementary Figure S4). We find that the Gal4 binding rate (Figure 4E and F) to H2A–H2B proximal hexasomes (*k*_on hex_ = 0.0052 ± 0.0005 s^−1^nM^−1^) is ∼2-fold lower than nucleosomes (*k*_on nuc_ = 0.011 ± 0.004 s^−1^ nM^−1^). In contrast, the dissociation rate of Gal4 from H2A–H2B proximal hexasomes (*k*_off hex_ = 0.37 ± 0.05 s^−1^) and nucleosomes (*k*_off nuc_ = 0.32 ± 0.03 s^−1^) were nearly identical (Figure 4F and G). These Gal4 dissociation rates are significantly faster than from its consensus sequence within nucleosomes (55), which can be explained by the ∼100-fold higher affinity of Gal4 to its consensus binding site relative to the 2C sequence. We separately compared the ensemble FRET and smFRET measurement of the fraction of Gal4 bound hexasomes and nucleosomes (Supplementary Figure S5). We find that they agree, which implies that the surface tethering does not impact the smFRET measurements. These results imply that the 2-fold decrease in hexasome accessibility is due to a change in the Gal4 binding rate. Since the binding rate is influenced by the unwrapping/rewrapping equilibrium of the hexasome on the H2A–H2B proximal side relative to the nucleosome, these results indicate that the hexasome unwrapping equilibrium is reduced 2-fold relative to nucleosomes.

### A 23 base pair section of the 601 sequence is responsible for the asymmetric deposition of the H2A–H2B heterodimer

**Figure 5.**
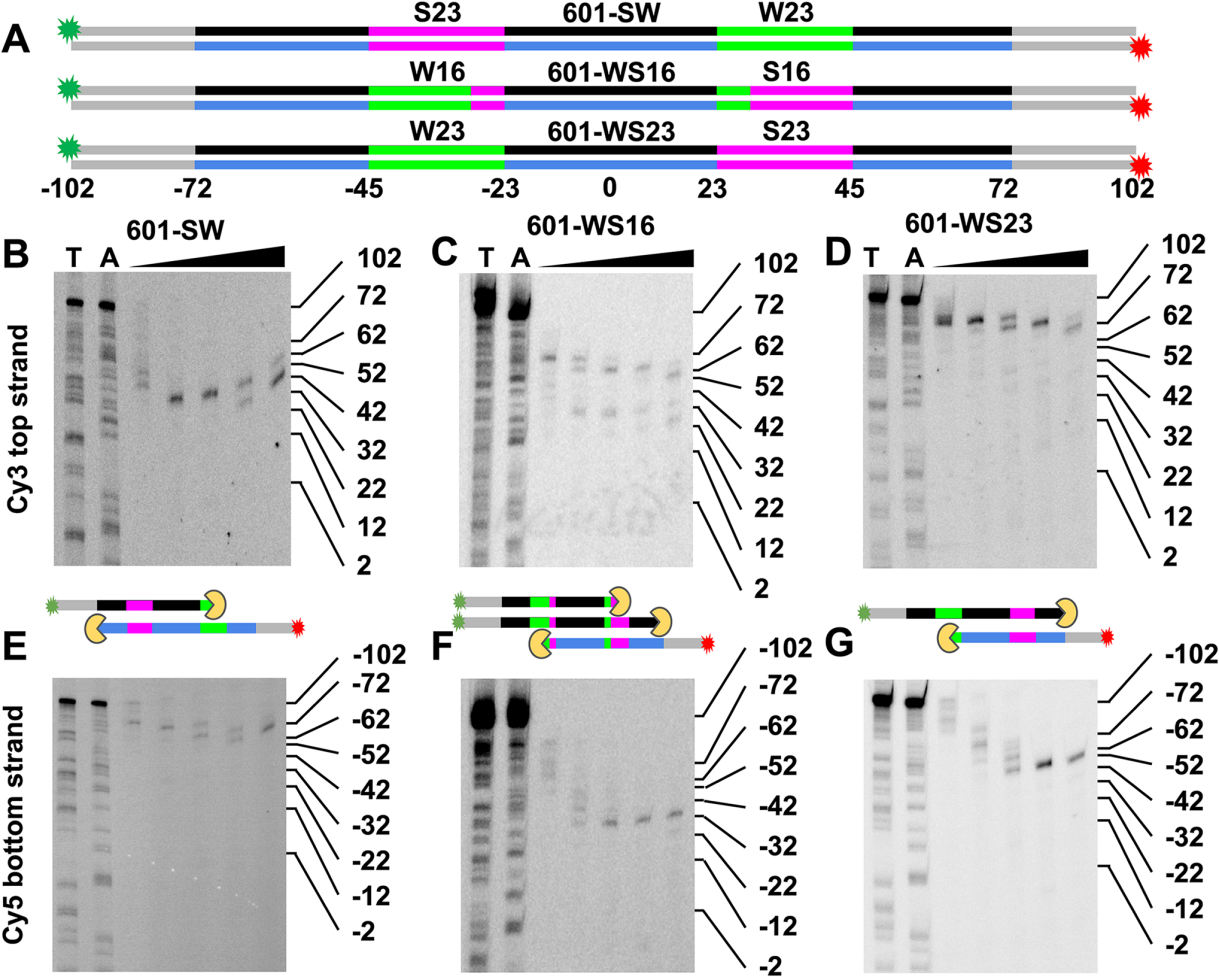
(**A**) Diagrams of the 601-SW, 601-WS16 and 601-WS23 DNA molecules used to determine the regions of the 601 NPS that are responsible for the asymmetric H2A–H2B binding. The 601-SW is the same molecule shown in Figure 2. The magenta S23 and green W23 regions are the original sequences in 601 NPS. Base pairs −45 to −30 were interchanged with 30 to 45 to create the 601-W chimera, and base pairs −45 to −23 were interchanged with 23 to 45 to create the 601-WS23 chimera. All three DNA molecules contain the Gal4 target sequence inserted at base pairs −65 to −46, but this is not highlighted. The top and bottom strands of the 601 sequence are shown in black and blue respectively, the 30 bp linker DNA is in gray, and Cy3 and Cy5 labels as green and red stars, respectively. (**B**–**D**) Cy3 images of 15% denaturing PAGE of ExoIII digested hexasomes containing 601-SW, 601-WS16 and 601-WS23, respectively. This visualizes the top DNA strand and indicates ExoIII stall sites on the right side of the dyad symmetry axis. Lanes T and A contain DNA sequencing ladders with ssDNA lengths terminated with a thymine or adenosine. The triangle indicates the lanes with ExoIII digested sample with 0.003, 0.01, 0.03, 0.1 and 0.3 units/μl of ExoIII for 5 min at 37°C. (**E**–**G**) Cy5 images of the same gels in B-D visualizing the bottom DNA strand. The diagrams between the Cy3 and Cy5 gels indicate the ExoIII (yellow) digestion stall positions.

The Bowman lab previously reported that the hexasomes reconstituted with 601 NPS are homogeneously oriented and positioned, where the H2A–H2B dimer is always bound to the same side of the 601 NPS (25). This combined with our observation that the 19 bp Gal4 DNA binding sequence can be inserted into the 601 NPS and not alter the formation of homogeneously positioned and oriented hexasomes indicates that the portion of the 601 sequence that is important for this property could be limited in length. To investigate this, we prepared 601 chimeras where portions of the 601 sequence that strongly (left) and weakly (right) bind the H2A–H2B dimer are replaced by the reverse complement of the same region on the opposite sides of the 601 NPS (Figure 5A). We reconstituted and sucrose gradient purified hexasomes with these 601 chimeras and then used ExoIII mapping to determine if the changes in the DNA sequence altered the hexasome position and orientation.

To design the 601 chimeras, we focused on the 601 sequence that is more than 26 base pairs into the nucleosome (Supplementary Table S1) since substituting the Gal4 binding site at base pairs −45 to −63 did not alter the preferential H2A–H2B binding to the left side of the 601 NPS (Figure 2). We first prepared the 601-W DNA (Figure 5A, Supplementary Table S1), which has the left 601 base pairs, −30 to −45, interchanged with the reverse complement of the right 601 base pairs, 30 to 45. ‘WS’ indicates that the left and right portions of the DNA sequence is designed to have Weak and Strong H2A–H2B affinity, respectively, which is the reverse of the original 601 sequence. These regions of the 601 DNA directly contact the H2A–H2B dimer and the inner side is located near where the ExoIII digestion stalls (Figure 2). We reconstituted hexasomes with 601-WS-16 DNA and determined the hexasome position with ExoIII mapping. The denaturing PAGE analysis of the digested DNA (Figure 5C, F) showed pause sites at 72 bases and 32 bases into the NPS on the right side and a pause site at −32 bases on the left side. This indicates that the H2A–H2B dimer binding preference was largely switched from the left to the right side by interchanging 601 base pairs −30 to −45 with 30–45.

To determine if we could fully switch the hexasome orientation, we prepared 601-WS23 (Figure 5A, Supplementary Table S1), a 601 chimera that interchanges the reverse complements of base pairs −23 to −45 and 23 to 45. This extends the interchanged region an additional 7 bp into the nucleosome and extends past the region of DNA that is in contact with the H2A–H2B dimer. However, this DNA region needs to bend to contact the H2A–H2B dimer and thus could impact dimer-DNA binding. We reconstituted and purified hexasomes that contain this DNA sequence and carried out ExoIII mapping. The results showed pause sites at 72 bases and −32 bases from the center of the 601 NPS (Figure 5D, G), indicating that the H2A–H2B dimer preference has completely switched orientation. To further confirm that this 23 base pair region of the 601 sequence is responsible for the asymmetric deposition of the H2A–H2B heterodimer, we prepared NPS with either both strong or both weak H2A–H2B binding sequences. This was done by replacing base pairs 23 to 45 with −23 to −45 or base pairs −23 to −45 with 23 to 45, to create 601-SS and 601-WW, respectively. We then prepared hexasomes with both of these DNA sequences and carried out ExoIII mapping. We find that hexasomes with either DNA sequence no longer have asymmetric ExoIII stall positions (Supplementary Figure S6) and thus no longer have preferential positioning of the H2A–H2B dimer. We therefore conclude that this 23 base pair section of the 601 sequence is fully responsible for the preferential H2A–H2B dimer binding.

### The TA dinucleotide repeats that are responsible for 601 nucleosome positioning are not fully responsible for the 601 asymmetric H2A–H2B dimer binding

The 601 NPS and the other NPSs isolated by Lowary and Widom contain a pattern of periodic TA/TT dinucleotides every 10 bases and CC/CG dinucleotides offset by 5 bases from this TA/TT pattern (56). This pattern contributes to the sequence's high affinity to the histone octamer, which appears to be due to their increase in the DNA’s flexibility and innate curvature (56). Interestingly, the 601 NPS contains this TA/TT and CC/CG pattern in the −23 to −45 base pair section of the 601 NPS, while it is absent in the 23–45 base pair section of the 601 NPS. This suggested to us that the TA/TT and CC/CG pattern could be responsible for the asymmetric H2A–H2B dimer binding of the 601 NPS.

To investigate if these dinucleotides alone account for the H2A–H2B dimer deposition asymmetry, we prepared four additional 601 chimeras: 601-WS-TA12, 601-WS-TA123, 601-WS-TA12-CC1 and 601-WS-TA123-CC12 (Supplementary Table S1). ‘TA’ indicates that the bases in phase with the TA/TT pattern in the 601 sequence were swapped, while ‘CC’ indicates that the bases in phase with the CC/CG pattern were swapped. The numbers 1, 2, and 3 indicate which of the first, second and third occurrence of this dinucleotide pattern within our 23 base pair regions were interchanged. We prepared sucrose gradient purified hexasomes with each of these 601 chimeras and mapped the hexosome position using ExoIII and denaturing PAGE (Supplementary Figures S7-S8). The hexasomes containing 601-WS-TA12, 601-WS-TA123 and 601-WS-TA12-CC1 had a minimal impact on the ExoIII stall positions. Additional minor stall sites were observed, which are consistent with a reduction in the preference of H2A–H2B location. The 601-WS-TA123-CC12 had the largest impact on the H2A–H2B binding location, where H2A–H2B appears to bind similarly on both sides of the dyad axis within hexasomes. Therefore, interchanging TA/TT and CC/CG dinucleotides reduces the H2A–H2B binding asymmetry, but did not reverse the H2A–H2B preferential binding as we observed for 601-WS-23. Together, these results indicate that the TA/TT and CC/CG positions contribute to H2A–H2B asymmetric binding. However, they do not alone determine the highly asymmetric H2A–H2B binding to the 601 NPS. Instead, the entire 23 base pair sequence within the 601 NPS appears to be important for the H2A–H2B dimer deposition asymmetry.

### The asymmetric hexasome formation is not influenced by the histone variant H2A.Z

The histone variant H2A.Z is 64% identical to H2A, located within actively transcribed genes (57), and structured within the nucleosome similarly to canonical H2A (58). We considered the possibility that H2A.Z-H2B dimers alter the asymmetric binding to the 601 NPS. We prepared hexasomes with H2A.Z and the 601-SW DNA, and then mapped their position with ExoIII digestions. Denaturing PAGE analysis of the ExoIII mapping shows that the hexasomes containing H2A.Z have the same position and orientation within the 601 NPS as those containing canonical H2A (Supplementary Figure S9). Therefore, the 601 NPS can also be used to prepare *in vitro* homogeneously oriented hexasomes containing H2A.Z.

### Hexasome orientation correlates with position within gene coding regions

Given our finding that H2A–H2B binds asymmetrically *in vitro*, we investigated where H2A–H2B asymmetry occurs *in vivo*. To this end, we used previously published ChIP-exo data from *S. cerevisiae* that determines the position of specific histones with base pair precision (26). From this data we determined H2A–H2B dimer positions and distinguished upstream and downstream biased hexasomes on a per gene basis. We explored heterodimer bias by investigating positional H2A–H2B dimer occupancy and correlations between dimer occupancies within a gene. In order to eliminate mispositioned and weakly positioned nucleosomes as well as any other experimental artifacts that would result in an apparent asymmetry of the entire nucleosome rather than an asymmetry in only the dimers, we limited ourselves to nucleosomal particles for which H4 occupancy was symmetrical. Within these, we distinguished between three different cases, upstream biased hexasomes, if the H2B signal was 2-fold higher for the upstream H2A–H2B dimer than for the downstream dimer, downstream biased hexasomes, if the reverse was true, and symmetric nucleosomes, if the signal for the two H2A–H2B dimers were less than 1.3-fold different (see Materials and Methods for details). We found that the fraction of upstream biased hexasomes, downstream biased hexasomes, and symmetric nucleosomes, at the first and third nucleosome position in a gene is significantly different from their distribution averaged over the first three nucleosome positions interrogated in (26) (*P*-values of 7 × 10^−12^ and 4 × 10^−6^ for the first and third nucleosome within a gene, respectively, Supplementary Table S4). We find nucleosomal particles with downstream H2A–H2B dimer preference are over-represented at the +1 position, and symmetric nucleosomal particles are over-represented at the +3 position. The second nucleosome position in a gene did not show significant differences in this distribution from the average distribution of all three positions. Interestingly, since the ratio of hexasomes to nucleosomes at the +3 position is likely closer to this ratio throughout the gene body, both +1 and +2 positions are likely over-represented with hexasomes relative to nucleosomes in the gene body as a whole.

Next, we investigated if there is a correlation between gene expression and hexasome orientation, as might be expected if RNA polymerase translocation causes asymmetric H2A–H2B dimer eviction, but did not find any evidence of such an effect (Supplementary Table S5). We also investigated the correlations between hexasome orientations at different positions within the same gene but did not find any to be significant (Bonferroni corrected *P* > 0.05, Supplementary Table S6). This is in apparent contrast to the findings in (26), where it was found that nucleosomal particles with upstream heterodimer preference tend to be followed by nucleosomal particles with downstream heterodimer preference. A major difference between our analysis and the one in (26) is that we limit ourselves to nucleosomal particles with a symmetric H4 signal. This excludes potential artifacts due to nucleosomal particles that do not even generate a symmetric signal for the tetramer but also reduces statistical power. Indeed, if we eliminate the constraint on the symmetry of the H4 signal, we do find that if a heterodimer is missing on one side of a nucleosome, the adjacent heterodimer in the neighboring nucleosome is also likely to be missing, which is consistent with the findings in (26) (*P*-values of 0.0008 and 0.0002 for the +1 and +2, and the +2 and +3 position in a gene, respectively, see Supplementary Table S7 for all *P*-values). The above findings of the differences between hexasomes and nucleosomes at the +1 and +3 positions remains significant under these relaxed conditions at *P*-values of 2 × 10^−17^ and 7 × 10^−9^, respectively (Supplementary Table S8). Overall, we conclude that the statistically different distributions of hexasome orientations at the first and third nucleosome position of a gene in *S. cerevisiae* indicate that hexasome orientation in the initial nucleosomes in *S. cerevisiae* is not random and thus likely related to biological function.

### DNA sequence correlates with hexasomes orientation within gene coding regions

**Figure 6.**
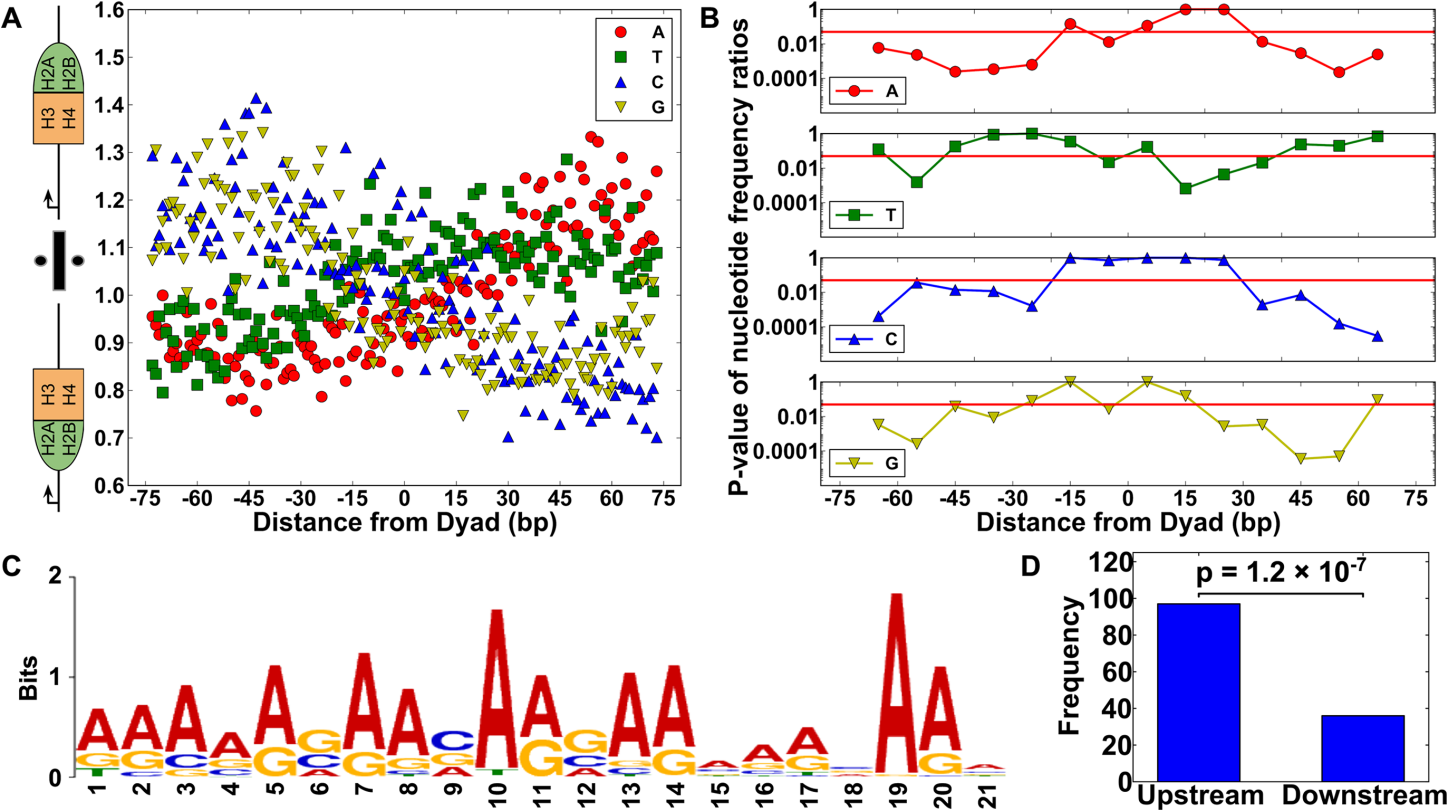
(**A**) Nucleotide frequency ratios of upstream vs. downstream biased hexasomes (shown in cartoon). A (blue circles) and T (green squares) are over-represented in regions missing an H2A–H2B heterodimer, while C (red up triangles) and G (yellow down triangles) are over-represented in regions with an H2A–H2B heterodimer present. (**B**) Bonferroni corrected *P*-values for the nucleotide frequency ratios obtained by *t*-tests of groups of 10 nucleotide positions compared against a null hypothesis of 1 for all ratios. (**C**) An example of a downstream binding motif identified as a weak binding motif by its significant *P*-value when the number of upstream and downstream occurances of the motif were compared in a binomial test as shown in (**D**).

Our combined observations that a 23 bp region of nucleosomal DNA can induce asymmetric H2A–H2B binding *in vitro* and that +1 hexasomes are biased where the H2A–H2B dimer is positioned in the downstream direction of the gene suggested to us that the underlying genome sequence could influence the hexasome orientation *in vivo*. To investigate this we again relied on the previously published ChIP-exo data (26) but in addition to extracting the presence and orientation of hexasomes, we collected their underlying sequences by mapping to a reference genome. By aggregating the combined sequences for upstream and downstream biased hexasomes, we obtained positional nucleotide distributions that give the percent probability that a nucleotide A, T, C or G, appears at a certain distance from the dyad separately for upstream biased (Supplementary Figure S10A) and downstream biased (Supplementary Figure S10B) hexasomes. Supplementary Figure S10C shows these frequencies for (symmetric) nucleosomes as a comparison. To determine if there is a difference in sequence composition depending on the orientation of the hexasome, we took the ratios of the upstream biased and downstream biased hexasome nucleotide distributions, which removed other types of sequence biases including those contained in gene coding regions (Figure 6A). These ratios show that C and G nucleotides are more likely at locations where a H2A–H2B heterodimer is present and A and to some extent T is more likely in locations where the heterodimer is missing. While we cannot plot a corresponding ratio for symmetric nucleosomes since this by definition equals unity, we can calculate *P*-values for whether the ratios of the upstream and downstream biased nucleosomes are significantly different from one. The *P*-values for the significance of these deviations from one are given in Figure 6B and confirm that the differences observed in Figure 6A are significant for all nucleotides but T except for the immediate vicinity of the dyad. We similarly find that the dinucleotides AA, TT, GG and GC are also correlated to hexasome orientation (Supplementary Figure S11). These results suggest that the underlying DNA sequence influences the formation and orientation of hexasomes within budding yeast gene coding regions.

### Specific DNA sequence motifs correlate with hexasome orientation

Next, we considered the possibility that the nucleotide and dinucleotide biases that correlate with hexasome orientation are due to underlying sequence motifs. By grouping sequences based on downstream biased hexasomes (Supplementary Figure S12), upstream biased hexasomes (Supplementary Figure S13), and nucleosomes (Supplementary Figure S14), we determined sequence motifs present within each sequence group using MEME (46). After removing motifs also found in unbiased nucleosomes (Supplementary Figure S14), we found five motifs that are specifically associated with upstream and downstream biased hexasomes, which are indicated by an asterisk (*) in Supplementary Figures S12 and S13. We next quantified the frequency with which the motif is found on the upstream and downstream side of the hexasome (Supplementary Figures S12 and S13). We find that for all five of these sequences the upstream vs. downstream positioning is significantly asymmetric, which supports the idea that these motifs influence hexasome orientation. Figure 6C shows one such motif. According to Figure 6D it is enriched in the upstream half of downstream biased hexasomes, leading us to conclude that it is a weak binding motif (see also Supplementary Figure S12A). By similar reasoning, we find that 3 of the five motifs are weak binding, while the other two are strong binding (Supplementary Figure S12 and S13). These results indicate that DNA sequences in upstream and downstream biased hexasomes are significantly different from each other, which can be captured in specific sequence motifs and suggests that specific DNA sequences could help establish and orient hexasomes at the beginning of gene coding regions.

### DNA sequences based on in vivo hexasome motifs influence hexasome orientation in vitro

Our observation that there are sequence motifs within *S. cerevisiae* genes that are correlated with hexasome orientation *in vivo* suggested to us that we could use these motifs to prepare homogeneously oriented hexasomes *in vitro*. First, we investigated if the 601 NPS, which fully orients hexasomes, contains at least one of the motifs we identified above. Using FIMO (48), we found that none of the identified motifs are contained within the 601 NPS at the default *P*-value cutoff of 0.0001. This indicates that the sequence motifs found to be associated with hexasome orientation *in vivo* are distinct from the 23 bp sequence that determines hexasome orientation in the 601 NPS.

**Figure 7.**
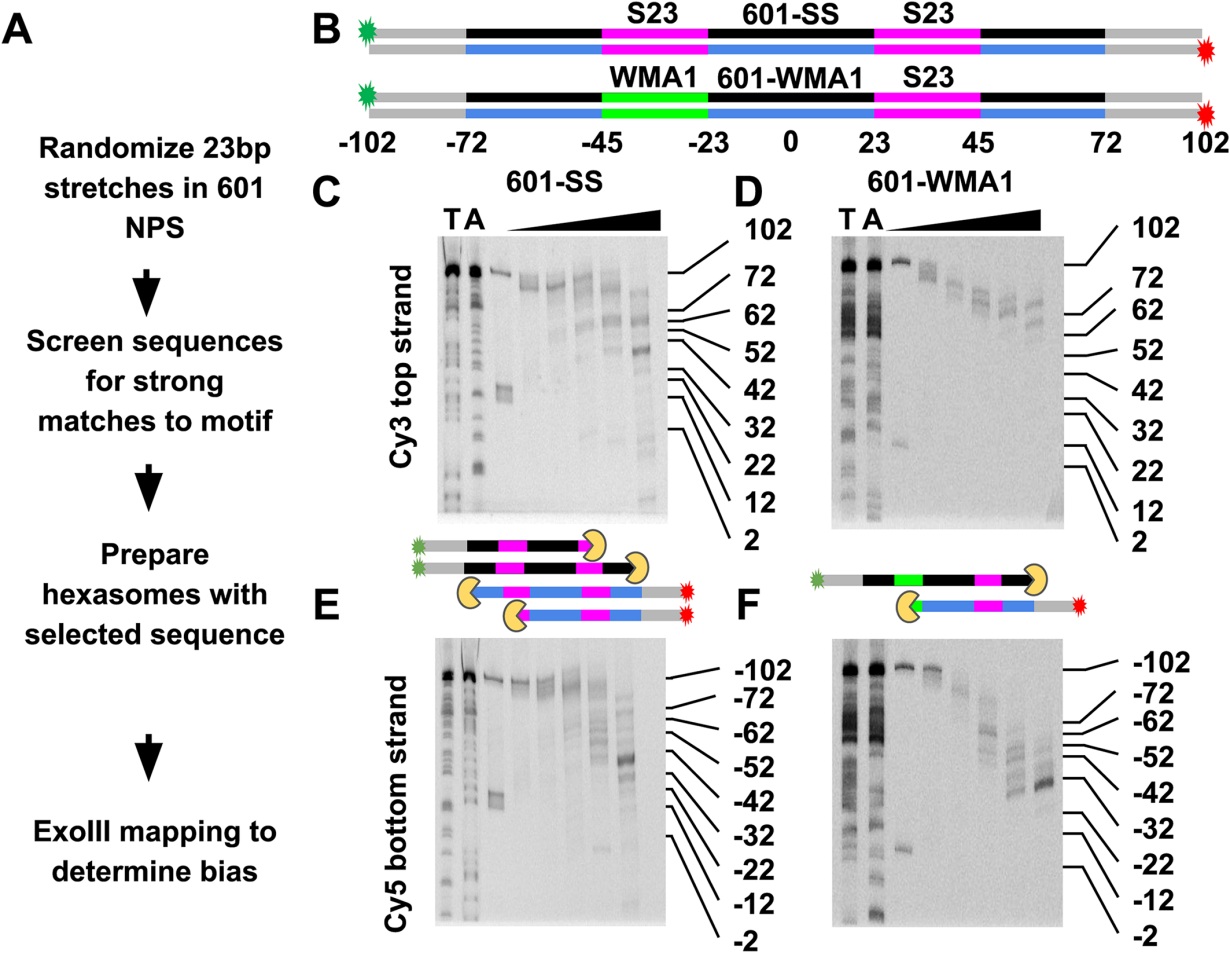
(**A**) Flow chart on how the 601 NPS was modified to contain the DNA sequence motifs that were identified to be correlated with hexasome orientation. (**B**) Diagrams of the 601-SS, and 601-WMA1 DNA molecules used to investigate if the WMA1 DNA sequence motif influences hexasome orientation. The 601-SS is the same molecule shown in Supplementary Figure S6. The magenta and green regions indicate DNA sequences that bind H2A–H2B dimers strongly and weakly, respectively. Both DNA molecules contain the Gal4 target sequence inserted at base pairs −65 to −46, but this is not highlighted. The top and bottom strands of the 601 sequence are shown in black and blue respectively, the 30 bp linker DNA is in grey, and Cy3 and Cy5 labels as green and red stars, respectively. (**C** and **D**) Cy3 Images of 15% denaturing PAGE of ExoIII digested hexasomes containing 601-SS and 601-WMA1, respectively. This visualizes the top DNA strand and indicates ExoIII stall sites on the right side of the dyad symmetry axis. Lanes T and A contain DNA sequencing ladders with ssDNA lengths terminated with a thymine or adenosine. The triangle indicates the lanes with ExoIII digested sample with 0.003, 0.01, 0.03, 0.1, and 0.3 units/μl of ExoIII for 5 minutes at 37°C. (**E** and **F**) Cy5 images of the same gels in B–D visualizing the bottom DNA strand. The diagrams between the Cy3 and Cy5 gels indicate the ExoIII (yellow) digestion stall positions.

We next decided to investigate if three of these motifs influence hexasome orientation. We chose two weak binding motifs: WMA (Figure 6C and Supplementary Figure S12A) and WMT (Supplementary Figure S12B), and one strong binding motif: SMC (Supplementary Figure S12C). These were chosen based on the *P*-value of the motif, the motif's total frequency, and the *P*-value of the asymmetry in the upstream and downstream motifs. We inserted the weak motifs WMA and WMT into the 601-SS NPS, and the strong motif SMC into the 601-WW NPS. As discussed above, both the 601-SS and the 601-WW do not influence the hexasome orientation, so before the insertion of the motif, the NPS does not orient the hexasome (Supplementary Figure S6). To determine specific sequences to study *in vitro*, we generated 10,000 601-like sequences where random sequences replaced the relevant 23 bp nucleosomal DNA region that we identified as influencing hexasome orientation. We selected two sequences each that best match one of the three selected specific motifs (see Figure 7A and Materials and Methods). This resulted in 6 sequences: 601-WMA1, 601-WMA2, 601-WMT1, 601-WMT2, 601-SM1 and 601SM2, which were prepared as DNA constructs with 30 bp extensions and labeled at opposite 5′ ends with Cy3 and Cy5. We reconstituted and purified hexasomes (Supplementary Figure S3) with these six DNA sequences and a H2A–H2B dimer to H2-H4 tetramer ratio of 1 to 1. At higher dimer to tetramer ratios, nucleosomes form as we observed with the 601 NPS (Figure 1B). We then mapped hexasome positions with ExoIII and find that 601-WMA1 (Figure 7D, F), 601-WMA2 (Supplementary Figure S15D, G), 601-WMT1 (Supplementary Figure S16C, F) and 601-WMT2 (Supplementary Figure S16D, G) all result in a switch from symmetric oriented to asymmetric oriented hexasomes, while 601-SMC1 and 601-SMC2 did not introduce asymmetric oriented hexasomes (Supplementary Figure S17). This indicates that some of the DNA sequence motifs we identified to be correlated with asymmetrically oriented hexasomes *in vivo* (WMA and WMT) cause the assembly of asymmetric hexasomes *in vitro*. This supports the conclusion that DNA sequence is influencing the orientation of hexasomes within gene coding regions in *S. cerevisiae*.

## DISCUSSION

In this study, we quantitatively investigated how DNA sequence influences the formation of hexasomes, and the DNA accessibility within hexasomes relative to both nucleosomes and DNA. Combined, these studies provide insight into how hexasomes may function *in vivo*. We had anticipated that DNA accessibility on the H2A–H2B distal side of the hexasome would be significantly higher relative to nucleosomes given that the ExoIII stall position is 30–40 base pairs into the nucleosome. But, we also hypothesized that this DNA region would remain less accessible than fully exposed DNA because of the positively charged histone tails and exposed regions of the histone octamer that could compete with TF binding. However, our results show that these histone domains do not reduce accessibility and that the DNA on the distal side of the hexasome is maximally exposed. The LexA occupancy at this target site location within the nucleosome is reduced by 10^5^ (55) relative to fully exposed DNA. This implies that the accessibility is increased by orders of magnitude on the H2A–H2B distal side of the hexasome after conversion from a nucleosome. *In vivo* there are many TF binding sites within the first 30 base pairs on either side of the nucleosome (59,60), the interconversion between a hexasome and nucleosome will dramatically impact TF occupancy within this H2A–H2B distal 30 base pair DNA region of the nucleosome.

Previous experiments using a combination of the LexA TF and restriction enzymes probed the impact of cooperative binding at opposite sides of the nucleosome and found that binding to a site within the entry-exit region of the nucleosome did not result in a measurable influence on binding to sites on the opposite side of the dyad (61). However, a more recent force spectroscopy study reported that unwrapping the DNA from one side of the nucleosome with an applied force stabilized the DNA wrapped on the opposite side of the nucleosome (35). Here, we find that 30–40 base pairs of DNA on the H2A–H2B distal side is completely unwrapped. This suggests that a hexasome is similar to a nucleosome partially unwrapped by 30–40 base pairs. Our finding that Gal4 binding within the H2A–H2B proximal side is reduced by 2.4-fold is consistent with the Ngo *et al.* (35) finding that nucleosomal DNA wrapping is stabilized by DNA unwrapping on the opposite side of the nucleosome. This 2.4-fold decrease in binding is similar to the resolution of the Moyle-Heyrman *et al.* study (61), and therefore might be why this study did not detect an impact of DNA unwrapping on one side of the nucleosome stabilizing DNA wrapping on the opposite side of the nucleosome.

The 2-fold change in DNA accessibility reported here is similar to the 2- to 3-fold changes previously reported to be induced by single histone PTMs and amino acid substitutions (32,37,40,62). Interestingly, combinations of PTMs can have a multiplicative impact on accessibility (40), and result in over an order of magnitude change in accessibility. This suggests that the removal of a H2A–H2B dimer in combination with other factors such as the addition or removal of histone PTMs could combine to have a much larger impact on DNA accessibility. Furthermore, the 2-fold change in DNA accessibility observed here could itself be biologically relevant for gene expression as we are reminded by dosage compensation and haploinsufficiency diseases. Finally, amino acid substitutions that influence DNA accessibility on a scale similar to our 2-fold change do not alter the nucleosome high resolution crystal structures (63). This suggests that the hexasome structure on the H2A–H2B proximal side is similar to the nucleosome structure, which is consistent with the recently reported structure of an overlapping hexasome–nucleosome hybrid molecule (13).

The strong DNA–histone charge interactions that stabilize the wrapped DNA around the histone octamer are located at regions where the minor groove of the DNA faces the histone octamer (1) every 10 base pairs. The location of the 23 base pair region we identified that determines the asymmetric H2A–H2B binding within the hexasome includes two of these minor groove contacts 25 and 35 base pairs from the dyad symmetry axis. This region of the nucleosome has particularly strong contacts as detected by high resolution force spectroscopy DNA unzipping experiments (49), which implies that this region has a high free energy cost for DNA unwrapping relative to the DNA closer to the entry-exit region of the nucleosome (50). This force spectroscopy study also reported that the dwell times for disrupting these DNA–histone contacts are much longer on the left side of the 601 DNA than the right side, and this asymmetry was also reported by force-FRET measurements (35). These measurements are consistent with our observation that this region of the nucleosome is responsible for the strong H2A–H2B interactions on the left side of the 601 NPS relative to the right side, which indicates that the interactions that preferentially stabilize DNA wrapping also preferentially bind the H2A–H2B heterodimer.

The observation that the +1 position is enriched in hexasomes supports the idea that hexasomes are a mark of the beginning of genes (26). The formation of hexasomes at the +1 position could be directly due to RNA Pol II transcription through the nucleosome. However, RNA Pol II induces the downstream H2A–H2B dimer to dissociate (64), while we find that the upstream H2A–H2B dimer to be preferentially depleted at the +1 position. This combined with our observation that the level of transcription does not correlate with the hexasome enrichment suggests that RNA Pol II transcription is not directly responsible for upstream dimer depletion. Alternatively, chromatin remodeling could be responsible for H2A–H2B dissociation. For example, SWI/SNF and RSC, which are both targeted to promoters (65) are able to induce H2A–H2B dimer dissociation by sliding a nucleosome into an adjacent nucleosome (22). In addition, a more recent study of Chd1 remodeling of hexasomes reported that Chd1 slides hexasomes unidirectionally away from the dimer distal side of the hexasome (25). Therefore, the preferred orientation of downstream hexasomes would prevent chromatin remodelers such as Chd1 from sliding nucleosomes into the promoter region. Future *in vivo* studies should investigate functional connections between hexasome orientation and chromatin remodeling.

In addition to chromatin remodeling, it has been known for some time that poly-A and poly-T sequences are preferentially enriched in promoter regions that are depleted of nucleosomes (66). Furthermore, free energy measurements of poly-A and poly-T sequences show that these sequences have a low affinity to histone octamers (67). Our finding that the A-rich and T-rich sequence motifs we identified bind weakly to H2A–H2B dimers is consistent with previous studies of histone occupancy, and suggests that A-rich or T-rich ∼20 base pair stretches near the +1 nucleosome position could help with hexasome formation. Interestingly, we find that the +1 nucleosomes are more likely to contain an A-rich or T-rich sequence motif than the +2 or +3 nucleosomes (Supplementary Figure S18). Combined with our findings that hexasomes at the +1 position tend to be dimer downstream biased (Supplementary Table S4), these results suggest that A-rich and T-rich sequences that suppress nucleosome formation in promoters may also be involved in forming downstream oriented hexasomes in the +1 nucleosomes, which are located at the start codon and thus straddle the 3′ end of the promoter.

Finally, these *in vivo* hexasome analyses are derived from a large ensemble of cells and are based on the average occupancy of H2A–H2B dimers and H3–H4 tetramers. Therefore, these analyses will miss subpopulations and dynamic effects, which are likely important for how DNA sequence influences nucleosome and hexasome assembly, disassembly and function. Future studies on the dynamics of hexasome and nucleosome formation, interconversion and removal will be important to determine the full role of DNA sequence on the role of hexasomes in regulating transcription.

## Supplementary Material

gkz283_Supplemental_File
