# Supplementary material for: DNA sequence influences hexasome orientation to regulate DNA accessibility
Source: Nucleic Acids Res. 2019 Apr 24;47(11):5617–33. doi: 10.1093/nar/gkz283 (PMC6582347; doi:10.1093/nar/gkz283)
Supplement: gkz283_Supplemental_File [file gkz283_supplemental_file.pdf]

601-SW	5'-CTGGAGA**CCGGAGGGCTGCCCTCCGG**TCAATTGGTCGTAGACAGCTCTA<br>GCACCGCTTAAACGCACGTACGCGCTGTCCCCCGCGTTTTAACCGCCAA<br>GGGGATTACTCCCTAGTCTCCAGGCACGTGTCAGATATATACATCCTGT-3'
601-WS16	5'-CTGGAGA**CCGGAGGGCTGCCCTCCGG**CTGGAGACTAGGGAGTAGCTCTA<br>GCACCGCTTAAACGCACGTACGCGCTGTCCCCCGCGTTTTAACCGCCAA<br>GGGGATTGTCTACGACCAATTGAGCACGTGTCAGATATATACATCCTGT-3'
601-WS23	5'-CTGGAGA**CCGGAGGGCTGCCCTCCGG**CTGGAGACTAGGGAGTAATCCCC<br>GCACCGCTTAAACGCACGTACGCGCTGTCCCCCGCGTTTTAACCGCCAA<br>TAGAGCTGTCTACGACCAATTGAGCACGTGTCAGATATATACATCCTGT-3'
601-WS-TA12	5'-CTGGAGA**CCGGAGGGCTGCCCTCCGG**TTGATTGGTCGGGGACAGCTCTA<br>GCACCGCTTAAACGCACGTACGCGCTGTCCCCCGCGTTTTAACCGCCAA<br>GGGGATTACTTACTAGTCTCTGGGCACGTGTCAGATATATACATCCTGT-3'
601-WS-TA123	5'-CTGGAGA**CCGGAGGGCTGCCCTCCGG**TTGATTGGTCGGGGACAGCTCCC<br>GCACCGCTTAAACGCACGTACGCGCTGTCCCCCGCGTTTTAACCGCCAA<br>TAGGATTACTTACTAGTCTCTGGGCACGTGTCAGATATATACATCCTGT-3'
601-WS-TA12-CC1	5'-CTGGAGA**CCGGAGGGCTGCCCTCCGG**TTGATTACTCGGGGACAGCTCTA<br>GCACCGCTTAAACGCACGTACGCGCTGTCCCCCGCGTTTTAACCGCCAA<br>GGGGATTACTTACTACCCTCTGGGCACGTGTCAGATATATACATCCTGT-3'
601-WS-TA123-CC12	5'-CTGGAGA**CCGGAGGGCTGCCCTCCGG**TTGATTACTCGGGGACAACTCCC<br>GCACCGCTTAAACGCACGTACGCGCTGTCCCCCGCGTTTTAACCGCCAA<br>TAGGACTACTTACTACCCTCTGGGCACGTGTCAGATATATACATCCTGT-3'
601-SS:	5'-CTGGAGA**CCGGAGGGCTGCCCTCCGG**TCAATTGGTCGTAGACAGCTCTA<br>GCACCGCTTAAACGCACGTACGCGCTGTCCCCCGCGTTTTAACCGCCAA<br>TAGAGCTGTCTACGACCAATTGAGCACGTGTCAGATATATACATCCTGT-3'
601-WW	5'-CTGGAGA**CCGGAGGGCTGCCCTCCGG**CTGGAGACTAGGGAGTAATCCCC<br>GCACCGCTTAAACGCACGTACGCGCTGTCCCCCGCGTTTTAACCGCCAA<br>GGGGATTACTCCCTAGTCTCCAGGCACGTGTCAGATATATACATCCTGT-3'

Supplementary Table S1: The 147 base pair sequences of the 601 NPS chimeras. The Gal4 site is black and bold. The left and right 23 bp of 601 that asymmetrically bind H2A-H2B are in magenta and green, respectively.

Motif-weak-A-1	5’-CTGGAGA**CCGGAGGGCTGCCCTCCGG**TTCAAAAAGAAAAAAAATAGAGA GCACCGCTTAAACGCACGTACGCGCTGTCCCCCGCGTTTTAACCGCCAA TAGAGCTGTCTACGACCAATTGAGCACGTGTCAGATATATACATCCTGT-3’
Motif-weak-A-2	5’-CTGGAGA**CCGGAGGGCTGCCCTCCGG**GAAGAACGCAGCAGAAGGAACAA GCACCGCTTAAACGCACGTACGCGCTGTCCCCCGCGTTTTAACCGCCAA TAGAGCTGTCTACGACCAATTGAGCACGTGTCAGATATATACATCCTGT-3’
Motif-weak-T-1:	5’-CTGGAGA**CCGGAGGGCTGCCCTCCGG**TCCTTTCTGTTTTAATTTGGGGT GCACCGCTTAAACGCACGTACGCGCTGTCCCCCGCGTTTTAACCGCCAA TAGAGCTGTCTACGACCAATTGAGCACGTGTCAGATATATACATCCTGT-3’
Motif-weak-T-2	5’-CTGGAGA**CCGGAGGGCTGCCCTCCGG**TTAACTTTATCTTTCACTGTTAC GCACCGCTTAAACGCACGTACGCGCTGTCCCCCGCGTTTTAACCGCCAA TAGAGCTGTCTACGACCAATTGAGCACGTGTCAGATATATACATCCTGT-3’
Motif-strong-1:	5’-CTGGAGA**CCGGAGGGCTGCCCTCCGG**CTGGAGACTAGGGAGTAATCCCC GCACCGCTTAAACGCACGTACGCGCTGTCCCCCGCGTTTTAACCGCCAA CTATTTTCGCTACCATAGCCACCGCACGTGTCAGATATATACATCCTGT-3’
Motif-strong-2:	5’-CTGGAGA**CCGGAGGGCTGCCCTCCGG**CTGGAGACTAGGGAGTAATCCCC GCACCGCTTAAACGCACGTACGCGCTGTCCCCCGCGTTTTAACCGCCAA TTCTTCCTCAATTCGCTCTACATGCACGTGTCAGATATATACATCCTGT-3’

Supplementary Table S2: The 147 base pair sequences of the 601 NPS that contain motifs that were determined to have weak or strong H2A-H2B affinites. The Gal4 site is black and bold. The magenta and green sequences indicate if they are predicted to have strong and weak H2A-H2B binding affinities, respectively.

601-Gal4-S	[CY3] 5'-CTGGAGACCGGAGGGCTGCCCTCCGGTCAATTGG-3'<br>[Biotin] 5'-CGCATGCTGCAGACGCGTTACGTATCGG-3'
601-Gal4-W	[Cy3] 5'-ACAGGATCCGGAGGGCAGC-3'<br>[Biotin] 5'-TCTAGAGCTAGCCTAGGCTCGAGAAGCTTGTCGACG-3'
601-LexA-W	[Cy3] 5'-TTACTGTATGAGCATACAGTATGGAG-3'<br>[Biotin] 5'-TACGACTCACTATAGGGAAAGCTCGG-3'
DNA-LexA	[Cy3] 5'- ATACTGTATGAGCATACAGTACAATTGGTCGTAGCAAGCT-3'<br>[Biotin] 5'- CCCCTTGGCGGTTAAAACG[Cy5-aminoC6dT]GGGGGACAGC-3'
ExoIII mapping DNA	[CY3] 5'-TCATAAGGAGGACACTGGGACATGCATCGG-3'<br>[CY5] 5'-GTAGCGTCAACTCACTGCCCTATGCATTAT-3'

Supplementary Table S3. The oligonucleotide PCR primers for the DNA constructs

Numbers of upstream hexasomes, downstream hexasomes, and nucleosomes in all positions compared to numbers in:	
+1 position	7.4×10^{-12}
+2 position	0.056
+3 position	4.0×10^{-6}

Supplementary Table S4: P-values for distributions of upstream biased hexasomes, downstream biased hexasomes, and nucleosomes in each position (+1, +2, and +3) as compared to the overall distribution in all positions. We find downstream biased hexasomes over-represented at the +1 position, and nucleosomes over-represented at the +3 position.

Numbers of upstream hexasomes, downstream hexasomes, and nucleosomes compared to numbers in +1 position with:		Numbers of upstream hexasomes, downstream hexasomes, and nucleosomes compared to numbers in +2 position with:		Numbers of upstream hexasomes, downstream hexasomes, and nucleosomes compared to numbers in +3 position with:	
High expression	0.65	High expression	0.0073	High expression	0.81
Med expression	0.85	Med expression	0.15	Med expression	0.77
Low expression	0.47	Low expression	0.56	Low expression	0.80

Supplementary Table S5: Correlations between distribution of upstream, downstream, and unbiased hexasomes and gene expression for each position in a gene (+1, +2, and +3). Gene expression levels were determined using TFIIB occupancy group data. We find no significant differences (Bonferroni corrected p>0.05).

Upstream/Downstream Hexasome Correlations in Genes			
Up +1/Up +2	0.013	Down +1/Down +2	0.084
Up +1/Up +3	0.34	Down +1/Down +3	0.57
Up +2/Up +3	0.035	Down +2/Down +3	0.41
Up +1/Down +2	0.46	Down +1/Up +2	0.10
Up +1/Down +3	0.55	Down +1/Up +3	0.78
Up +2/Down +3	0.31	Down +2/Up +3	0.21

Supplementary Table S6: P-values for correlations between upstream or downstream biased hexasomes and position (+1, +2, or +3) within a gene. We did not find any significant correlations between orientations of different hexasomes within the same gene (Bonferroni corrected p>0.05).

Upstream/Downstream H2B Correlations in Genes			
Up +1/Up +2	0.48	Down +1/Down +2	0.11
Up +1/Up +3	0.054	Down +1/Down +3	0.72
Up +2/Up +3	0.012	Down +2/Down +3	0.0051
Up +1/Down +2	0.0008	Down +1/Up +2	0.95
Up +1/Down +3	0.087	Down +1/Up +3	0.20
Up +2/Down +3	0.0002	Down +2/Up +3	0.0029

Supplementary Table S7: P-values for correlations between upstream or downstream biased H2B occupancy and position (+1, +2, or +3) within a gene. If a heterodimer is missing on one side of a nucleosome, the adjacent heterodimer in the neighboring nucleosome is also likely to be missing, i.e., that nucleosomal particles with upstream heterodimer preference tend to be followed by nucleosomal particles with downstream heterodimer preference.

Numbers of upstream H2B, downstream H2B, and even H2B in all positions compared to numbers in:	
+1 position	2.1×10^{-17}
+2 position	0.011
+3 position	7.2×10^{-9}

Supplementary Table S8: P-values for distributions of nucleosomal particles with upstream heterodimer preferences, downstream heterodimer preferences, and no heterodimer preferences in each position (+1, +2, and +3) as compared to the overall distribution in all positions. We find nucleosomal particles with downstream heterodimer preference over-represented at the +1 position, and symmetric nucleosomal particles over-represented at the +3 position.

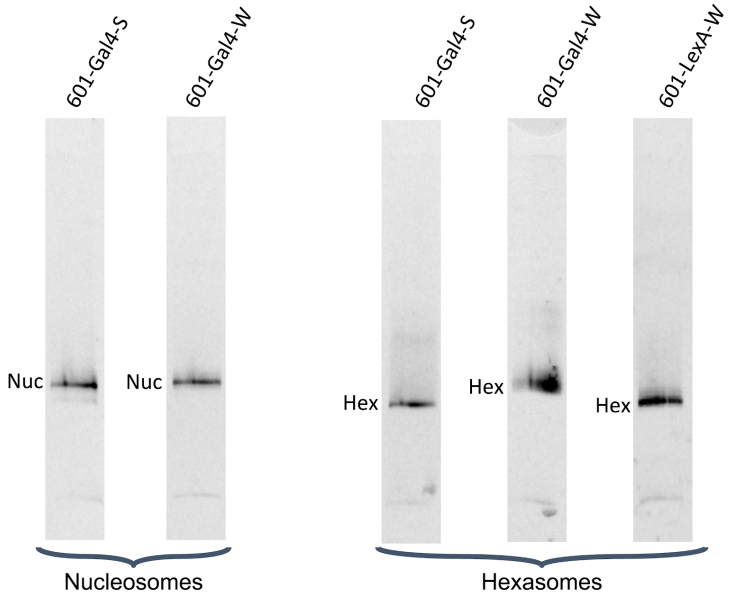

Supplementary Figure S1. Cy3 images of EMSA using native PAGE to analyze the purified nucleosome and hexasome samples used in the ensemble and single molecule FRET experiments.

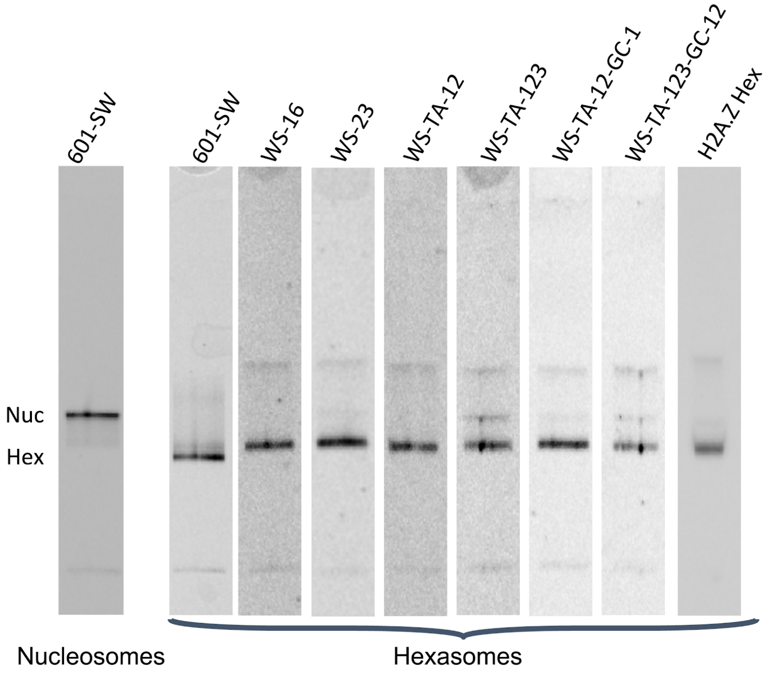

Supplementary Figure S2. Cy3 images of EMSA using native PAGE to analyze the purified nucleosome and hexasome samples used in the 601 chimera ExoIII mapping experiments.

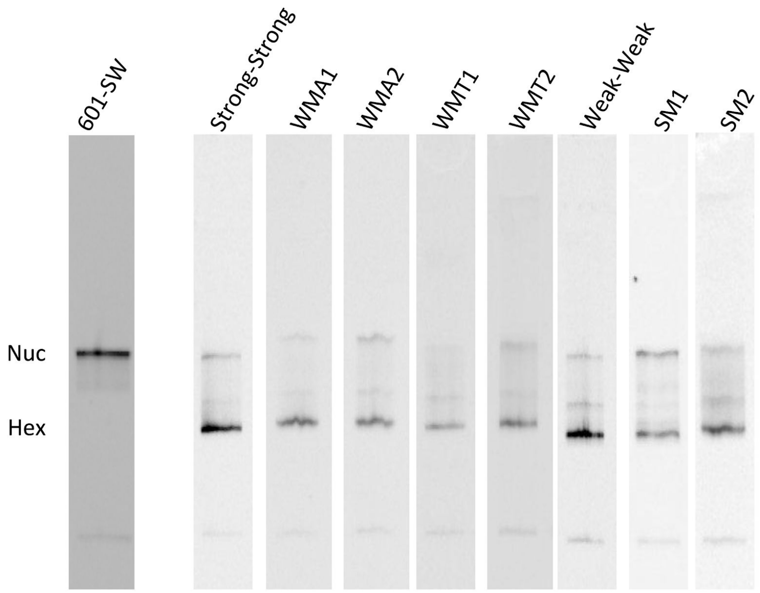

Supplementary Figure S3. Cy3 images of EMSA using native PAGE to analyze the purified nucleosome and hexasome samples used in the ExoIII mapping experiments with the DNA sequence motifs that are correlated with oriented hexasomes in gene coding regions.

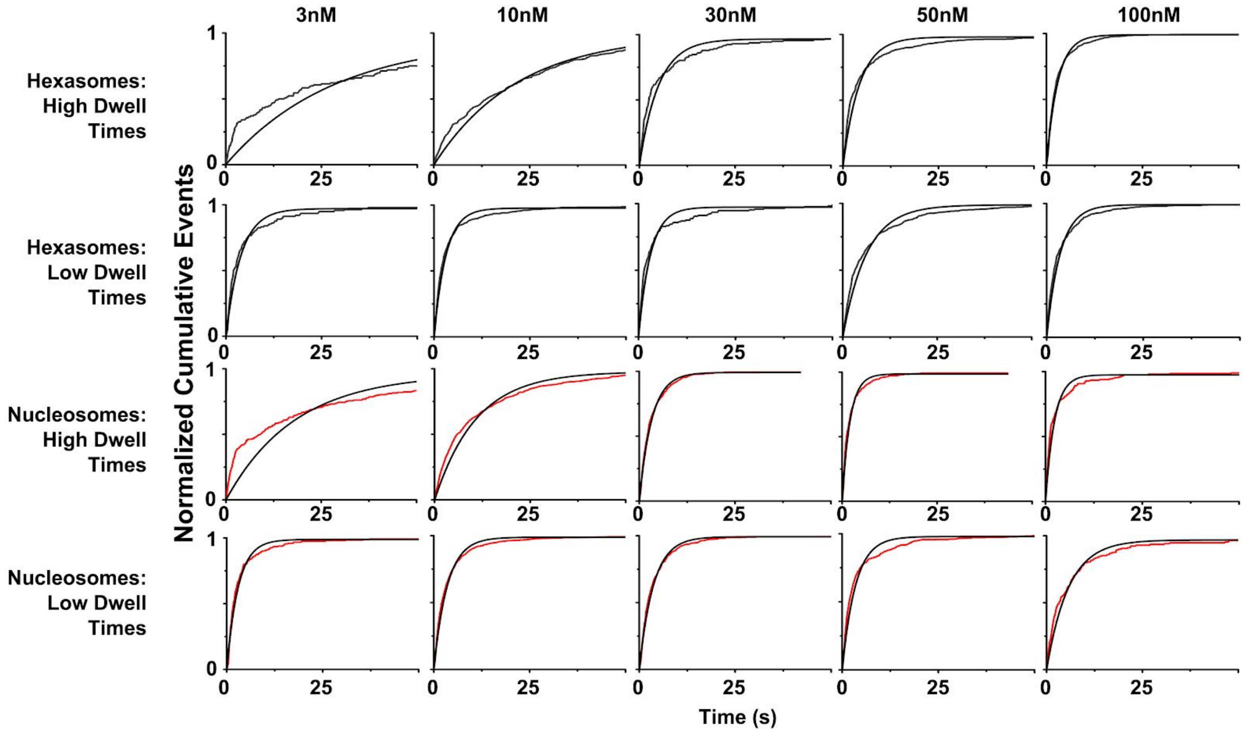

Supplementary Figure S4. Normalized cumulative distribution of FRET state dwell times for hexasomes (grey) and nucleosomes (red) at each Gal4 concentration measured. The first and third rows are dwell times in the high FRET state (Gal4 unbound). The second and fourth rows are dwell times in the low FRET state (Gal4 bound). Hexasomes and nucleosomes were prepared with 601-Gal4-S DNA (Figure 5A). Each distribution is fit (black) to the exponential distribution: $1 - \exp(-t/\tau)$, where τ is the characteristic dwell time.

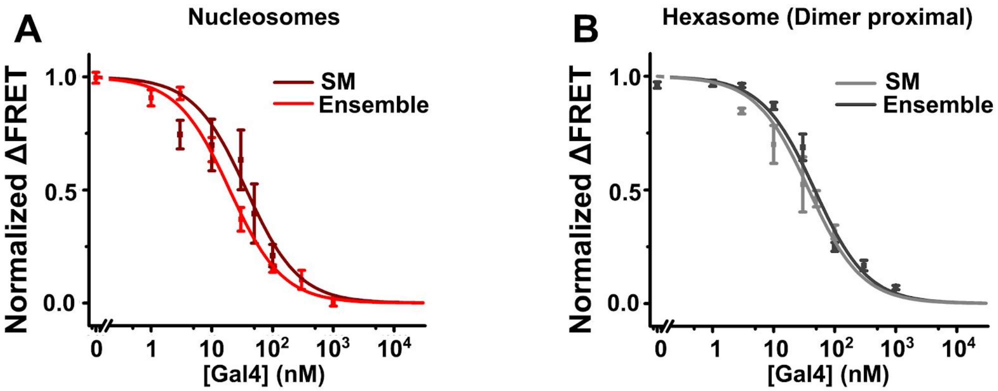

Supplementary Figure S5. (**A**) Normalized change in FRET with nucleosomes as a function of Gal4 concentration determined by single molecule measurements (red) and ensemble measurements (brown). (**B**) Normalized change in FRET with hexasomes where the H2A-H2B dimer is proximal to the Gal4 binding site determined by single molecule (grey) and ensemble measurements (black). The single molecule normalized change in FRET measurements were determined the average of the probability each nucleosome or hexasome was in the low FRET state and the uncertainty was determined from standard deviation of the mean. Each ensemble measurement was done in triplicate and the uncertainty was estimated from the standard deviation of the mean of each measurement. Each titration was fit to a non-cooperative binding isotherm.

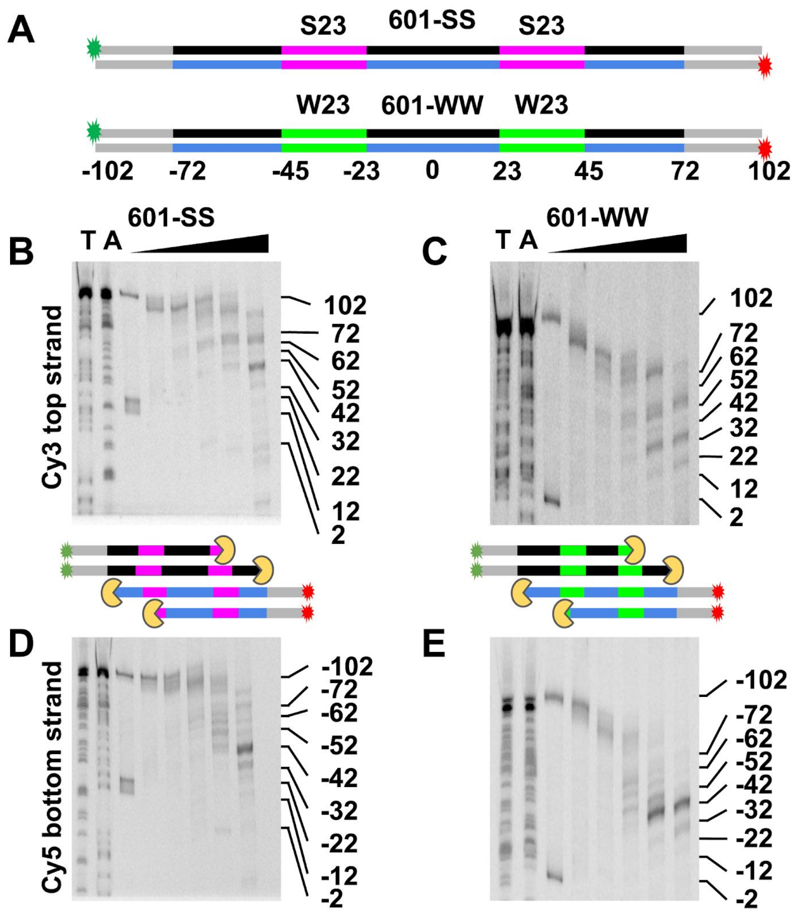

Supplementary Figure S6. (**A**) Diagrams of the 601-SS and 601-WW DNA molecules used to determine if these symmetric 601 chimeras contain asymmetrically oriented hexasomes. The magenta S23 and green W23 regions are the original strong and weak H2A-H2B binding sequences in the 601 NPS. Base pairs -45 to -23 were inserted into 23 to 45 to create the 601-SS chimera, while base pairs 45 to 23 were inserted into -23 to -45 to create the 601-WW chimera. Both DNA molecules contain the Gal4 target sequence inserted at base pairs -65 to -46, but this is not highlighted. The top and bottom strands of the 601 sequence are shown in black and blue respectively, the 30 bp linker DNA is in grey, and Cy3 and Cy5 labels as green and red stars, respectively. (**B**-**C**) Cy3 images of 15% denaturing PAGE of ExoIII digested hexasomes containing 601-SS and 601-WW, respectively. This visualizes the top DNA strand and indicates ExoIII stall sites on the right side of the dyad symmetry axis. Lanes T and A contain DNA sequencing ladders with ssDNA lengths terminated with a thymine or adenosine. The triangle indicates the lanes with ExoIII digested sample with 0.003, 0.01, 0.03, 0.1, and 0.3 units/μl of ExoIII for 5 minutes at 37 °C. (**D**-**E**) Cy5 images of the same gels in B-C visualizing the bottom DNA strand. The diagrams between the Cy3 and Cy5 gels indicate the ExoIII (yellow) digestion stall positions.

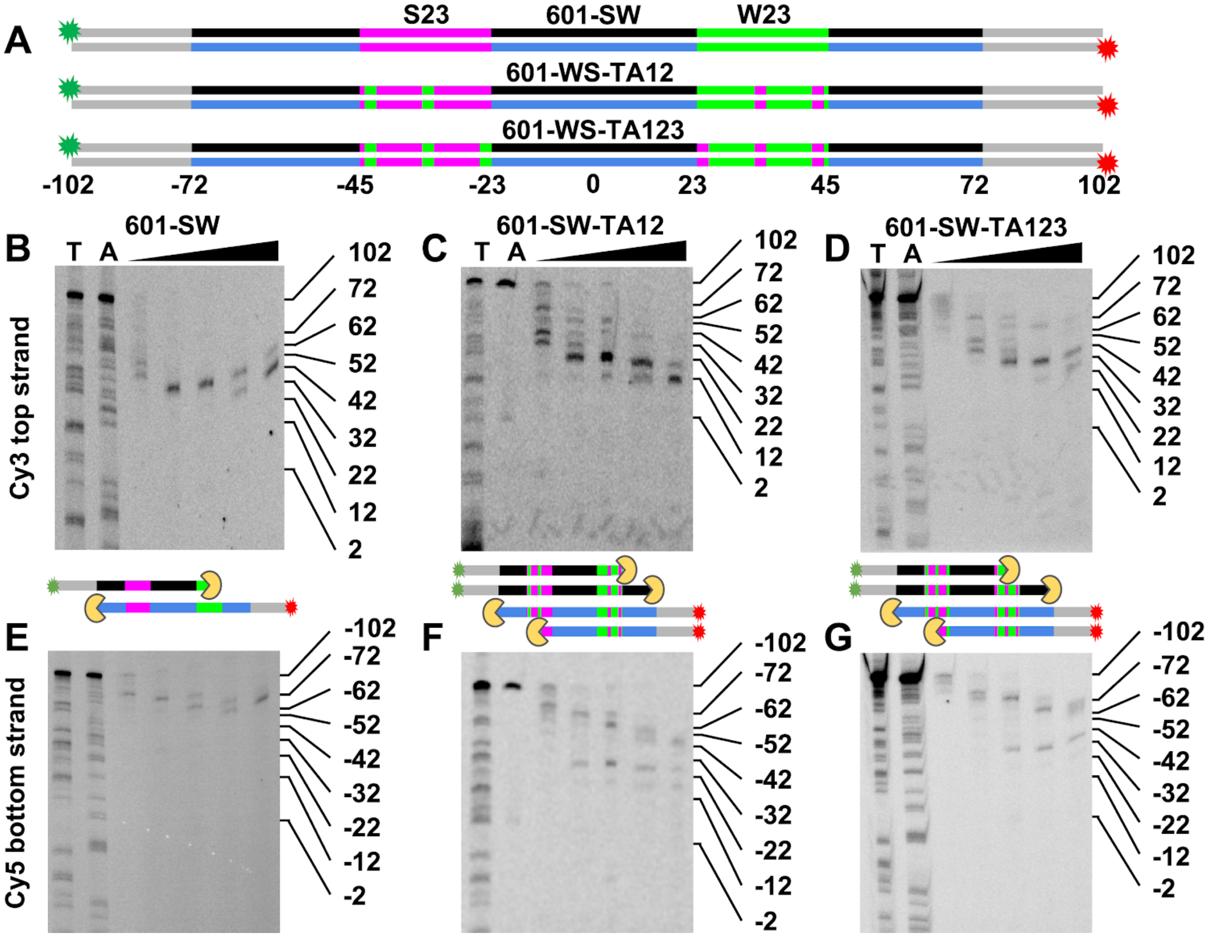

Supplementary Figure S7. (**A**) Diagrams of the 601-SW, 601-WS-TA12 and 601-WS-TA123 DNA molecules used to determine the regions of the 601 NPS that are responsible for the asymmetric H2A-H2B binding. The 601-SW is the same molecule as shown in Figure 2. The green S23 and magenta W23 regions are the original sequences in 601 NPS. Base pairs -44 to -43 were interchanged with 43 to 44, and -34 to -33 were interchanged with 33 to 34 to create the 601-WS-TA12 chimera. In addition to these changes, base pairs -24 to -23 were interchanged with 23 to 24 to create the 601-WS-TA123 chimera. All three DNA molecules contain the Gal4 target sequence inserted at base pairs -65 to -46, but this is not highlighted. The top and bottom strands of the 601 sequence are shown in black and blue respectively, the 30 bp linker DNA is in grey, and Cy3 and Cy5 labels are shown as green and red stars, respectively. (**B**-**D**) Cy3 images of 15% denaturing PAGE of ExoIII digested hexasomes containing 601-SW, 601-WS-TA12, and 601-WS-TA123, respectively. This visualizes the top DNA strand and indicates ExoIII stall sites on the right side of the dyad symmetry axis. Lanes T and A contain DNA sequencing ladders with ssDNA lengths terminated with a thymine or adenosine. The triangle indicates the lanes with ExoIII digested sample with 0.003, 0.01, 0.03, 0.1, and 0.3 units/μl of ExoIII for 5 minutes at 37 °C. (**E**-**G**) Cy5 images of the same gels in B-D visualizing the bottom DNA strand. The diagrams between the Cy3 and Cy5 gels indicate the ExoIII (yellow) digestion stall positions.

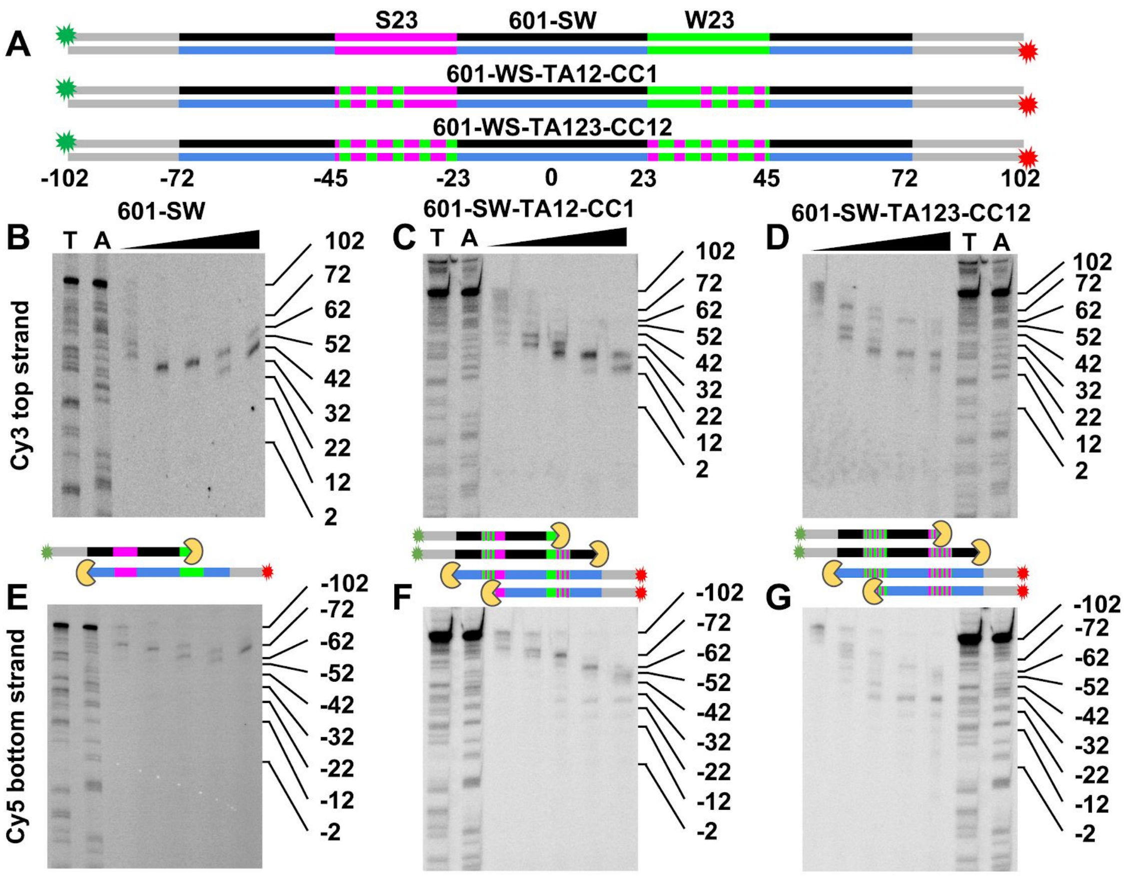

Supplementary Figure S8. (**A**) Diagrams of the 601-SW, 601-WS-TA12-CC1 and 601-WS-TA123-CC12 DNA molecules used to determine the regions of the 601 NPS that are responsible for the asymmetric H2A-H2B binding. The 601-SW is the same molecule as shown in Figure 2. The green S23 and magenta W23 regions are the original sequences in 601 NPS. Base pairs -44 to -43 were interchanged with 43 to 44, -40 to -39 were interchanged with 39 to 40, and -34 to -33 were interchanged with 33 to 34 to create the 601-WS-TA12-CC1 chimera. In addition to these changes, base pairs -30 to -29 were interchanged with 29 to 30, and -24 to -23 were interchanged with 23 to 24 to create the 601-WS-TA123-CC12 chimera. All three DNA molecules contain the Gal4 target sequence inserted at base pairs -65 to -46, but is not highlighted. The top and bottom strands of the 601 sequence is shown in black and blue respectively, the 30 bp linker DNA is in grey, and Cy3 and Cy5 labels as green and red stars, respectively. (**B**-**D**) Cy3 Images of 15% denaturing PAGE of ExoIII digested hexasomes containing 601-SW, 601-WS-TA12-CC1, and 601-WS-TA123-CC12, respectively. This visualizes the top DNA strand and indicates ExoIII stall sites on the right side of the dyad symmetry axis. Lanes T and A contain DNA sequencing ladders with ssDNA lengths terminated with a thymine or adenosine. The triangle indicates the lanes with ExoIII digested sample with 0.003, 0.01, 0.03, 0.1, and 0.3 units/µl of ExoIII for 5 minutes at 37 °C. (**E**-**G**) Cy5 images of the same gels in B-D visualizing the bottom DNA strand. The diagrams between the Cy3 and Cy5 gels indicate the ExoIII (yellow) digestion stall positions.

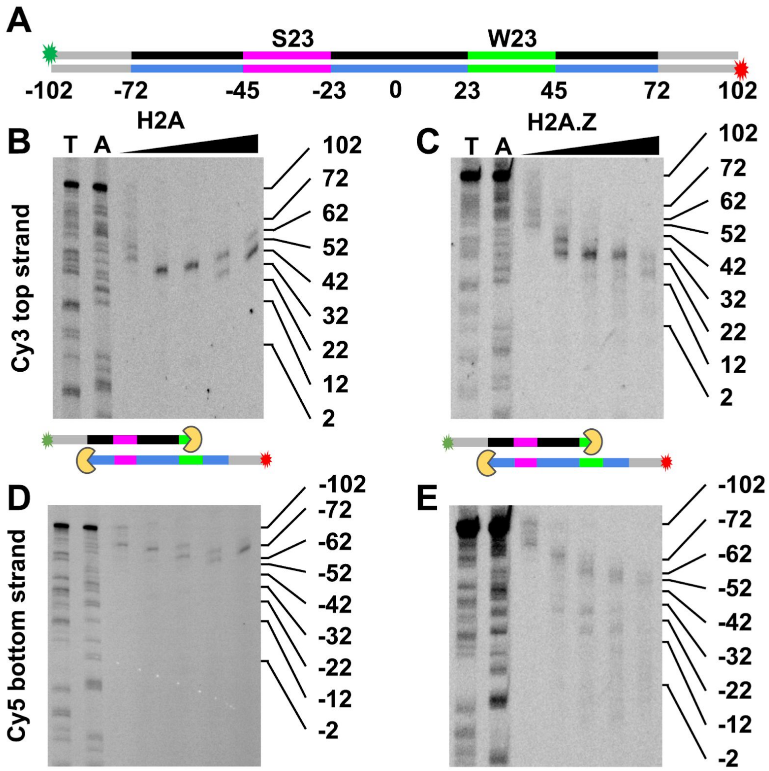

Supplementary Figure S9. (**A**) Diagram of the 601-SW DNA molecule, which is the same molecule as shown in Figure 2. The magenta S23 and green W23 regions are the original sequences in 601 NPS. (**B**-**C**) Cy3 Images of 15% denaturing PAGE of ExoIII digested 601-SW hexasomes containing either H2A or H2A.Z, respectively. This visualizes the top DNA strand and indicates ExoIII stall sites on the right side of the dyad symmetry axis. Lanes T and A contain DNA sequencing ladders with ssDNA lengths terminated with a thymine or adenosine. The triangle indicates the lanes with ExoIII digested sample with 0.003, 0.01, 0.03, 0.1, and 0.3 units/μl of ExoIII for 5 minutes at 37 °C. (**D**-**E**) Cy5 images of the same gels in B-D visualizing the bottom DNA strand. The diagrams between the nucleosome and hexasome gels indicate the ExoIII (yellow) digestion stall positions. The hexasomes stall positions are at -72 and +32.

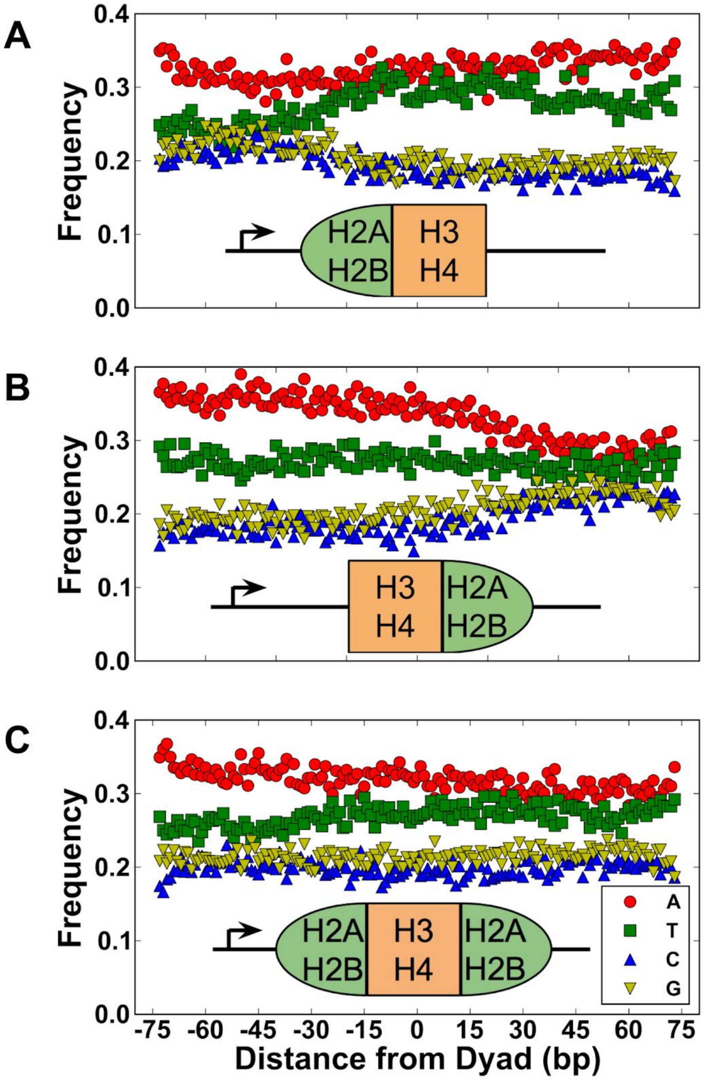

Supplementary Figure S10. Nucleotide frequencies in (**A**) upstream biased, (**B**) downstream biased, and (**C**) unbiased hexasomes obtained by counting the frequency of each nucleotide at each position relative to the dyad separately for nucleosomes and for upstream biased and downstream biased hexasomes. The frequencies in (**A**) and (**B**) were divided to create the ratios in **Figure 6A.**

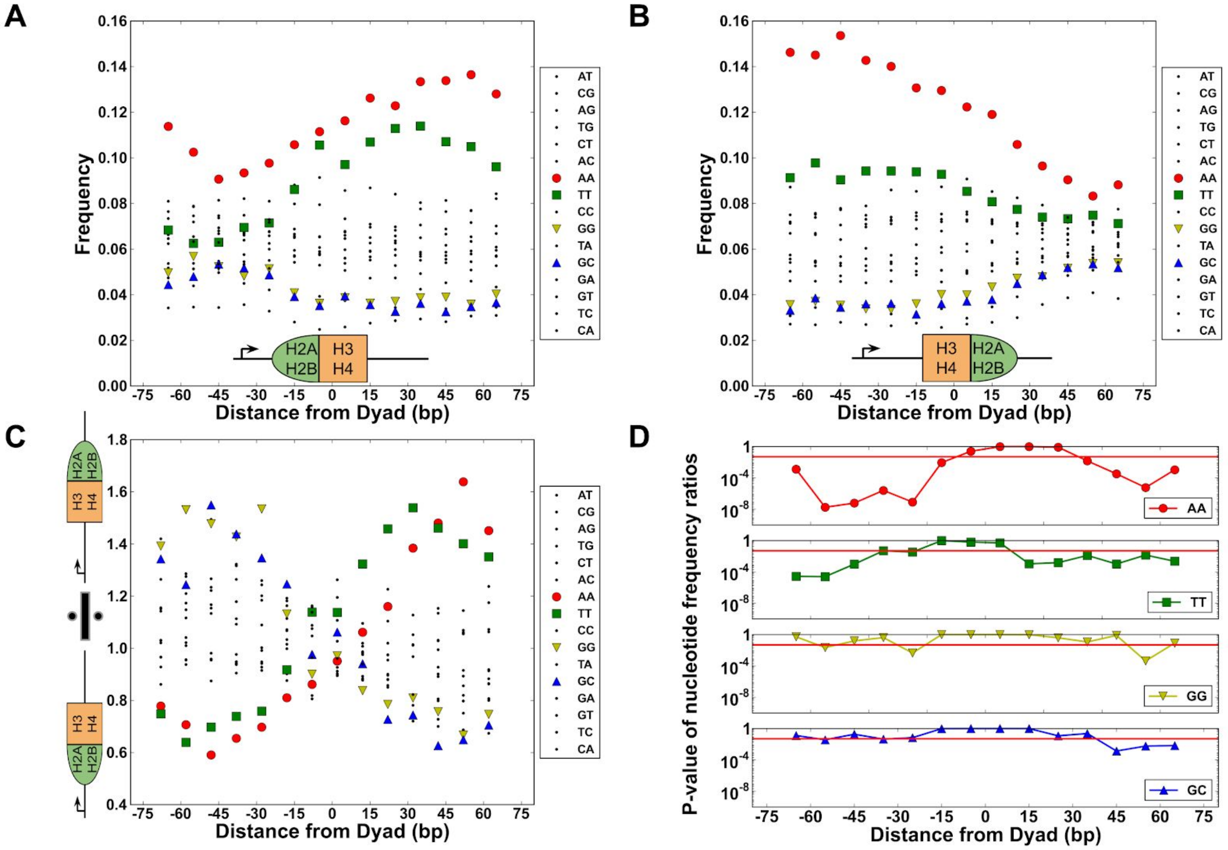

Supplementary Figure S11. Dinucleotide frequencies in (**A**) upstream and (**B**) downstream biased hexasomes obtained by counting the frequency of each dinucleotide at each position relative to the dyad separately for nucleosomes and for upstream biased and downstream biased hexasomes, and (**C**) their ratio. Significant dinucleotides (AA, TT, GG, GC) are highlighted with color. (**D**) Bonferroni corrected p-values for the dinucleotide frequency ratios obtained by t-tests of groups of 10 dinucleotide positions compared against a null hypothesis of 1 for all ratios.

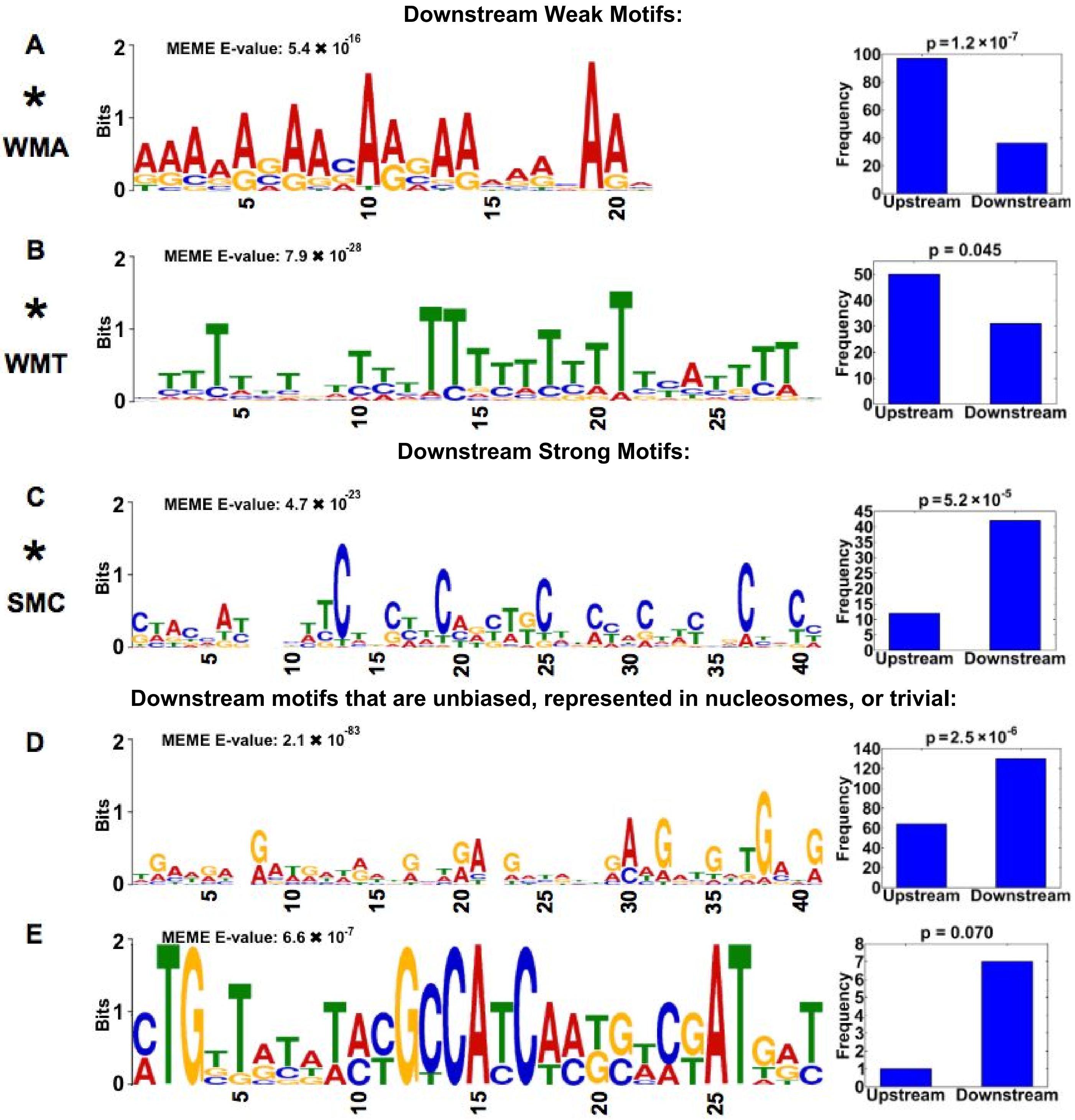

Supplementary Figure S12: Motifs identified by MEME in downstream biased hexasomes, MEME E-value describes the degree to which one finds these motifs in downstream biased hexasomes. Asterisks indicate motifs not found in nucleosomes. Bar graphs describe the center positions of these motifs within the hexasomes, and give a p-value for a binomial test of the asymmetry of the upstream/downstream distribution. We study the weak motifs WMA (A) and WMT (B), and the strong motif SMC (C).

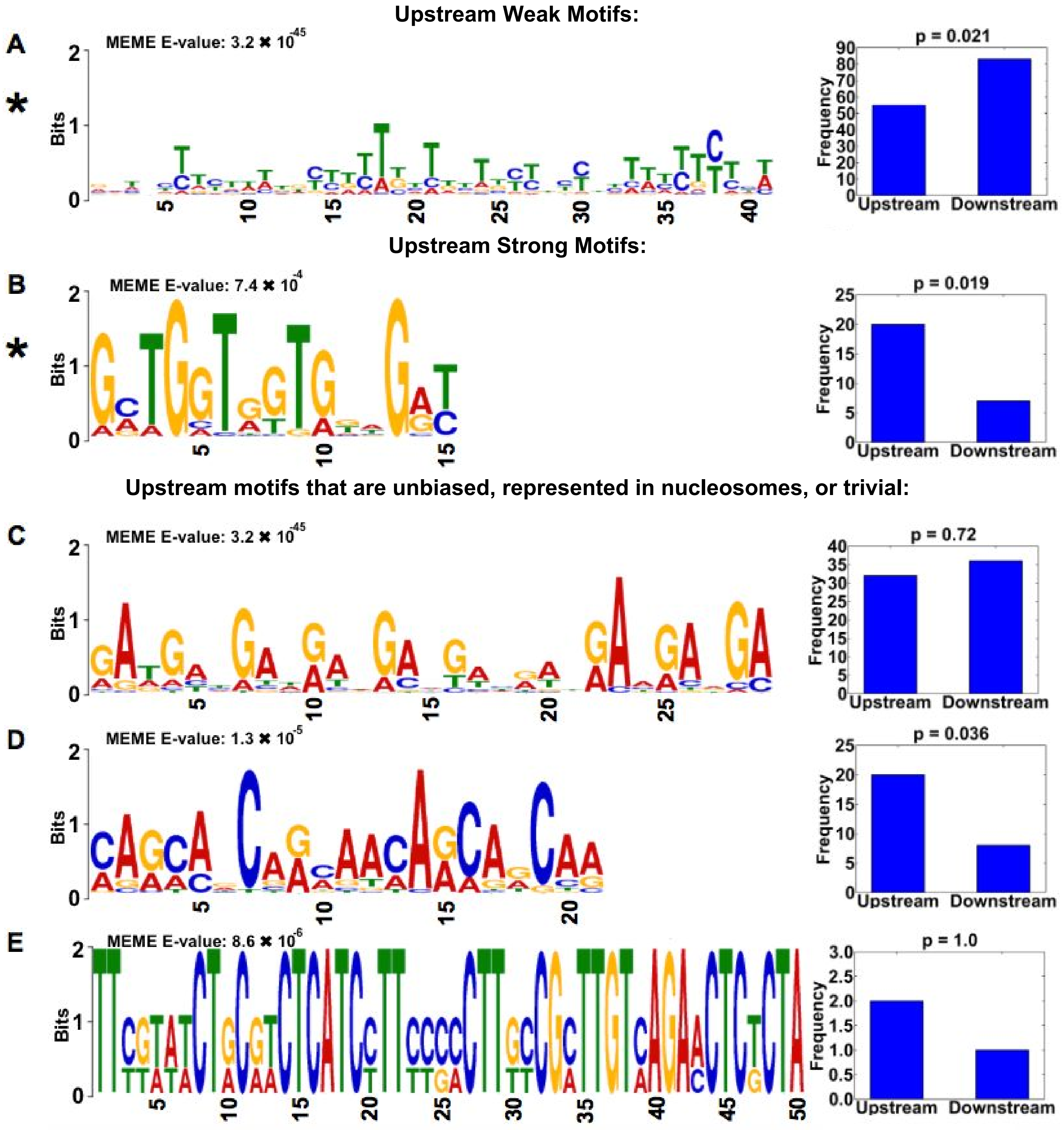

Supplementary Figure S13: Motifs identified by MEME in upstream biased hexasomes, MEME E-value describes the degree to which one finds these motifs in upstream biased hexasomes. Asterisks indicate motifs not found in nucleosomes. Bar graphs describe the center positions of these motifs within the hexasomes, and give a p-value for a binomial test of the asymmetry of the upstream/downstream distribution.

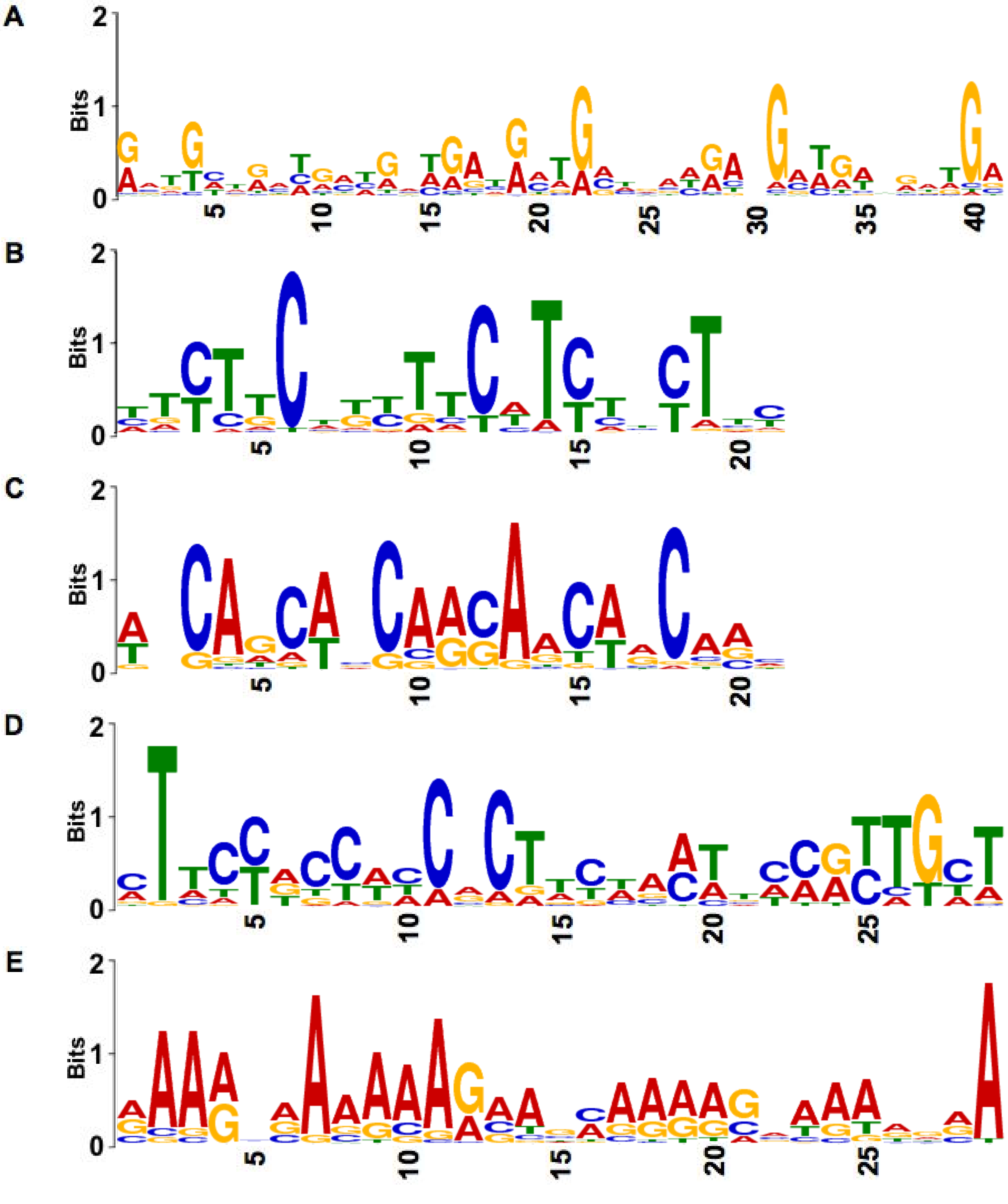

Supplementary Figure S14: Motifs identified by MEME in unbiased nucleosomes. Any hexasome motifs matching nucleosome motifs were ignored, in particular (A) invalidates Supplementary Figure (SF) 12D and SF13C, and (C) invalidates SF13D.

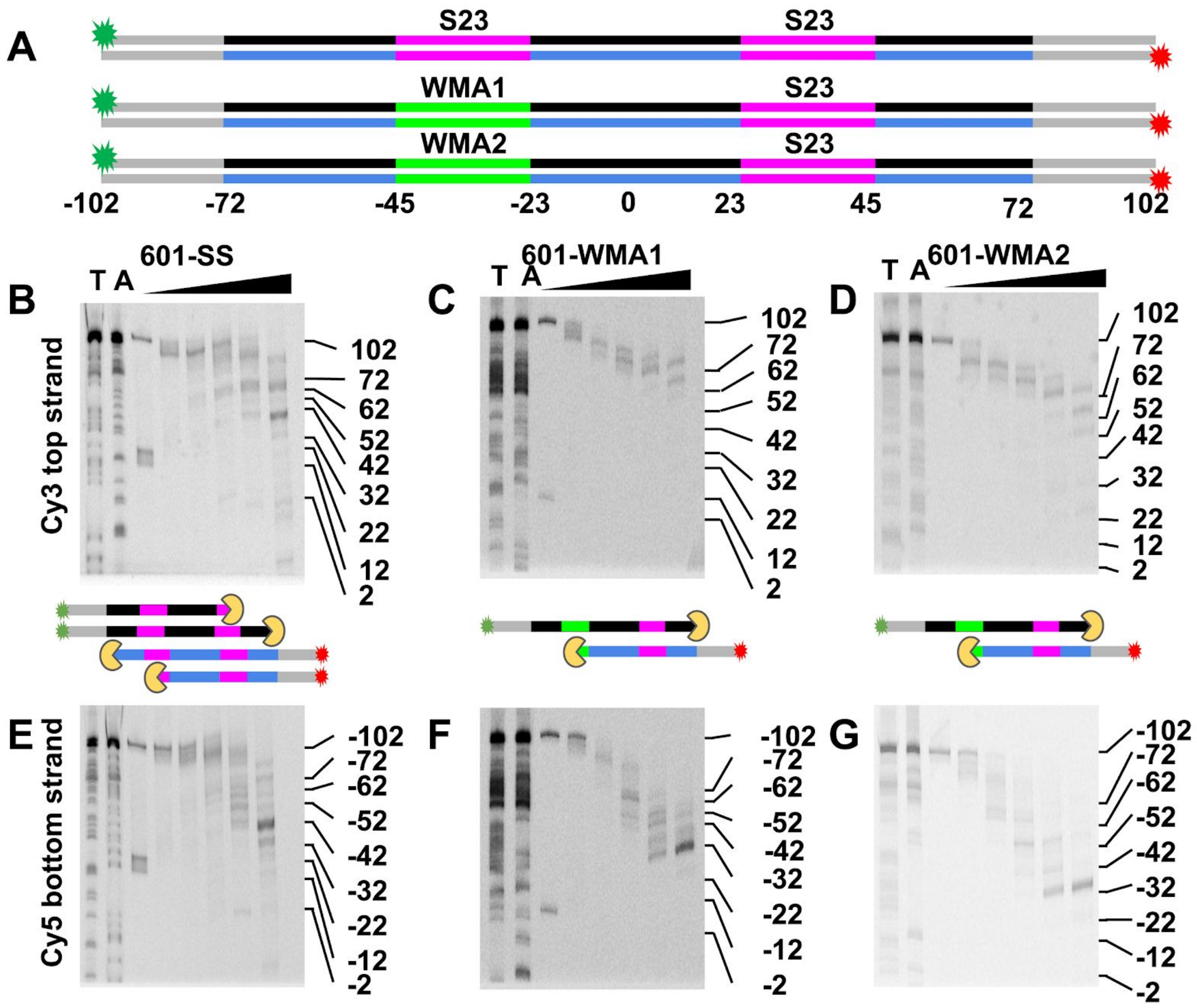

Supplementary Figure S15. (**A**) Diagrams of the 601-SS, 601-WMA1 and 601-WMA2 DNA molecules used to determine if the WMA sequence motif can orient hexasomes. The magenta region represents the 23 base pair sequence of the 601 NPS that strongly binds the H2A-H2B dimer. The green regions represent the two weak H2A-H2B binding sequences based on motif WMA (Figure 6C). Each DNA molecule contains the Gal4 target sequence inserted at base pairs -65 to -46, but this is not highlighted. The top and bottom strands of the 601 sequence are shown in black and blue respectively, the 30 bp linker DNA is in grey, and Cy3 and Cy5 labels as green and red stars, respectively. (**B**-**D**) Cy3 images of 15% denaturing PAGE of ExoIII digested hexasomes containing 601-SS, 601-WMA1 and 601-WMA2, respectively. This visualizes the top DNA strand and indicates ExoIII stall sites on the right side of the dyad symmetry axis. Lanes T and A contain DNA sequencing ladders with ssDNA lengths terminated with a thymine or adenosine. The triangle indicates the lanes with ExoIII digested sample with 0.003, 0.01, 0.03, 0.1, and 0.3 units/µl of ExoIII for 5 minutes at 37 °C. (**E**-**G**) Cy5 images of the same gels in B-D visualizing the bottom DNA strand. The diagrams between the Cy3 and Cy5 gels indicate the ExoIII (yellow) digestion stall positions.

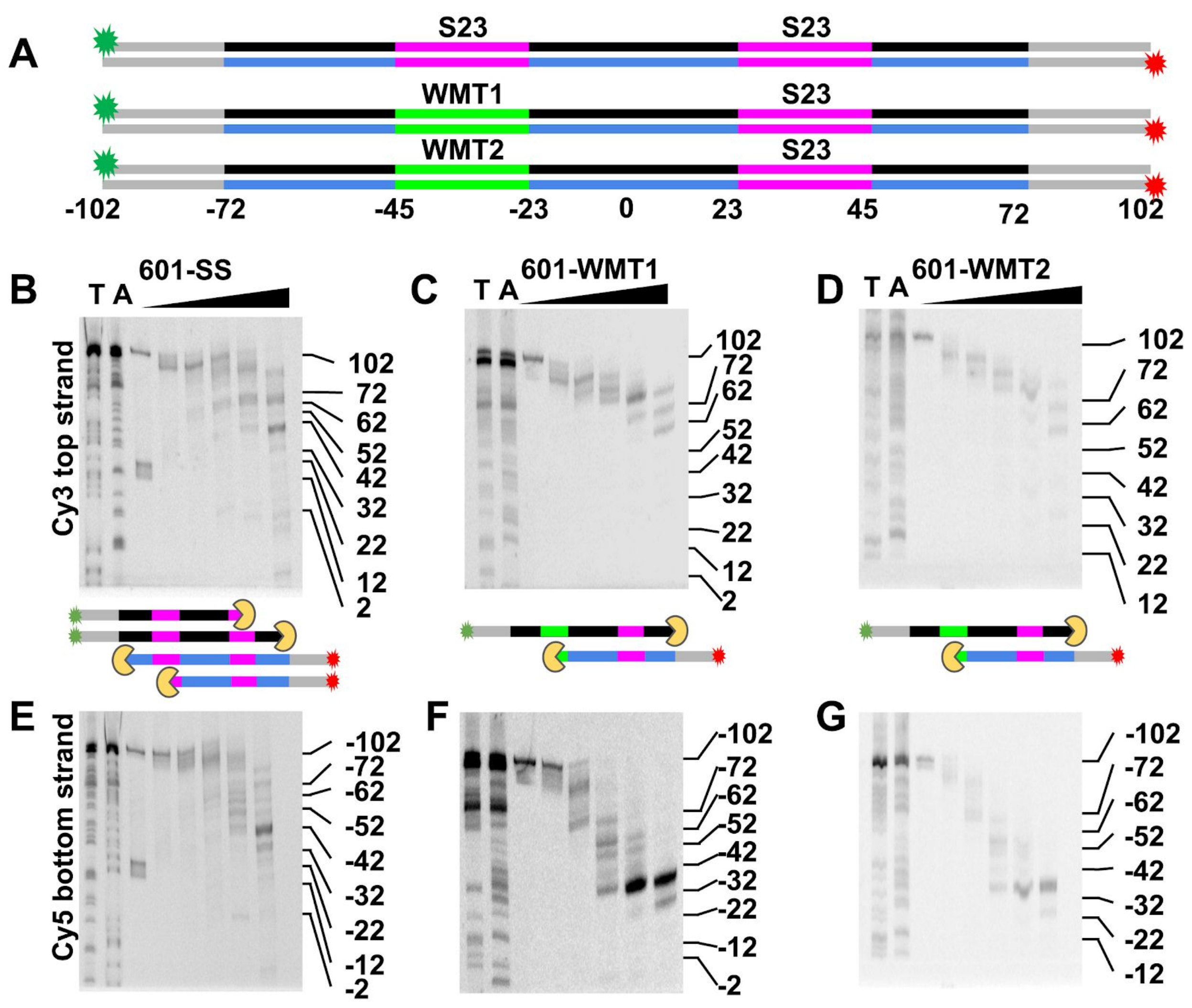

Supplementary Figure S16. (**A**) Diagrams of the 601-SS, 601-WMT1 and 601-WMT2 DNA molecules used to determine if the WMA sequence motif can orient hexasomes. The magenta region represents the 23 base pair sequence of the 601 NPS that strongly binds the H2A-H2B dimer. The green regions represent the two weak H2A-H2B binding sequences based on motif WMT (Supplementary Figure S12B). Each DNA molecule contains the Gal4 target sequence inserted at base pairs -65 to -46, but this is not highlighted. The top and bottom strands of the 601 sequence are shown in black and blue respectively, the 30 bp linker DNA is in grey, and Cy3 and Cy5 labels as green and red stars, respectively. (**B**-**D**) Cy3 images of 15% denaturing PAGE of ExoIII digested hexasomes containing 601-SS, 601-WMT1 and 601-WMT2, respectively. This visualizes the top DNA strand and indicates ExoIII stall sites on the right side of the dyad symmetry axis. Lanes T and A contain DNA sequencing ladders with ssDNA lengths terminated with a thymine or adenosine. The triangle indicates the lanes with ExoIII digested sample with 0.003, 0.01, 0.03, 0.1, and 0.3 units/μl of ExoIII for 5 minutes at 37 °C. (**E**-**G**) Cy5 images of the same gels in B-D visualizing the bottom DNA strand. The diagrams between the Cy3 and Cy5 gels indicate the ExoIII (yellow) digestion stall positions.

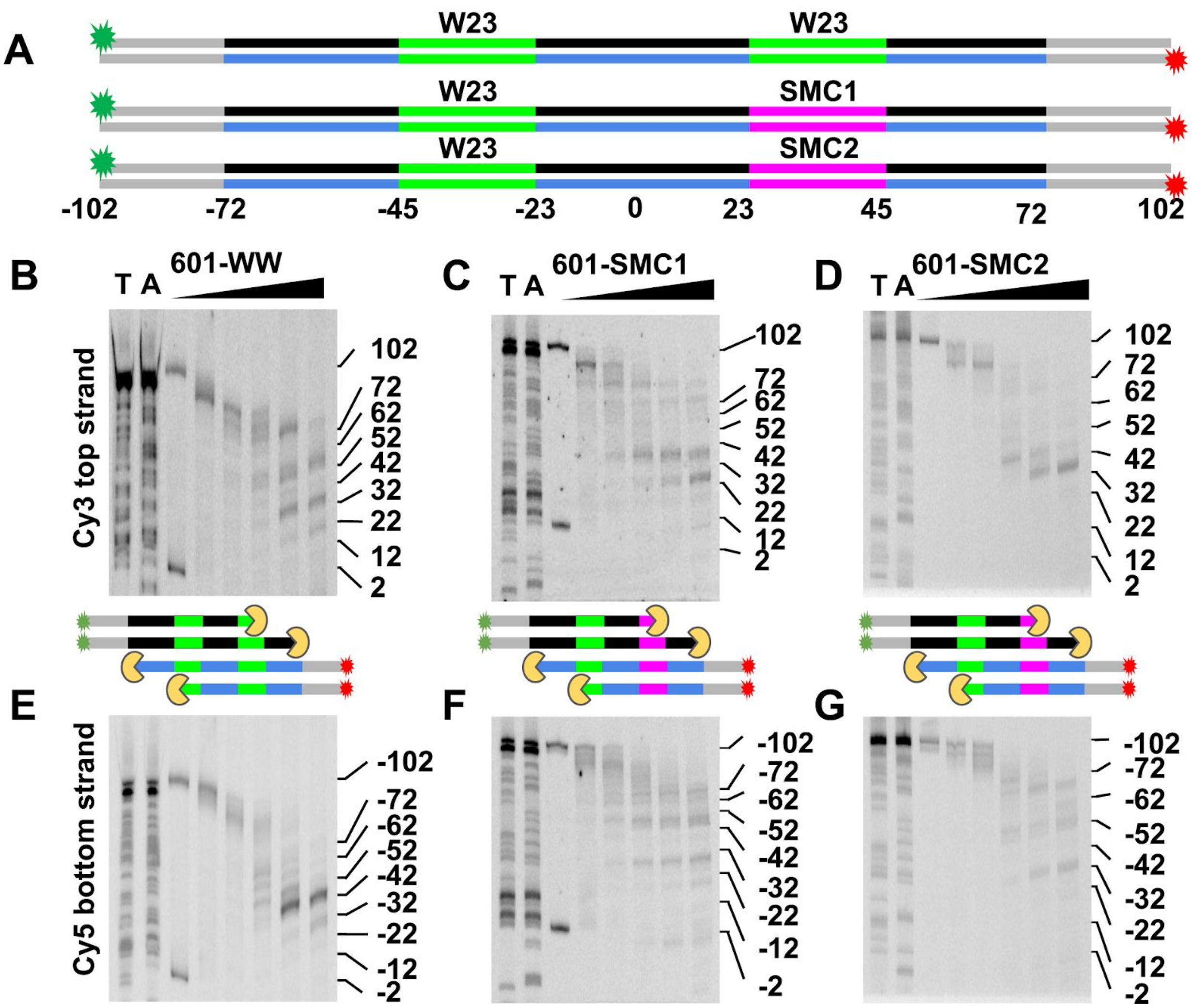

Supplementary Figure S17. (**A**) Diagrams of the 601-WW, 601-SM1 and 601-SM2 DNA molecules used to determine if the WMA sequence motif can orient hexasomes. The green region represents the 23 base pair sequence of the 601 NPS that weakly binds the H2A-H2B dimer. The magenta regions represent the two strong H2A-H2B binding sequences based on motif SM (Supplementary Figure S12C). Each DNA molecule contains the Gal4 target sequence inserted at base pairs -65 to -46, but this is not highlighted. The top and bottom strands of the 601 sequence are shown in black and blue respectively, the 30 bp linker DNA is in grey, and Cy3 and Cy5 labels as green and red stars, respectively. (**B**-**D**) Cy3 images of 15% denaturing PAGE of ExoIII digested hexasomes containing 601-WW, 601-SM1 and 601-SM2, respectively. This visualizes the top DNA strand and indicates ExoIII stall sites on the right side of the dyad symmetry axis. Lanes T and A contain DNA sequencing ladders with ssDNA lengths terminated with a thymine or adenosine. The triangle indicates the lanes with ExoIII digested sample with 0.003, 0.01, 0.03, 0.1, and 0.3 units/µl of ExoIII for 5 minutes at 37 °C. (**E**-**G**) Cy5 images of the same gels in B-D visualizing the bottom DNA strand. The diagrams between the Cy3 and Cy5 gels indicate the ExoIII (yellow) digestion stall positions.

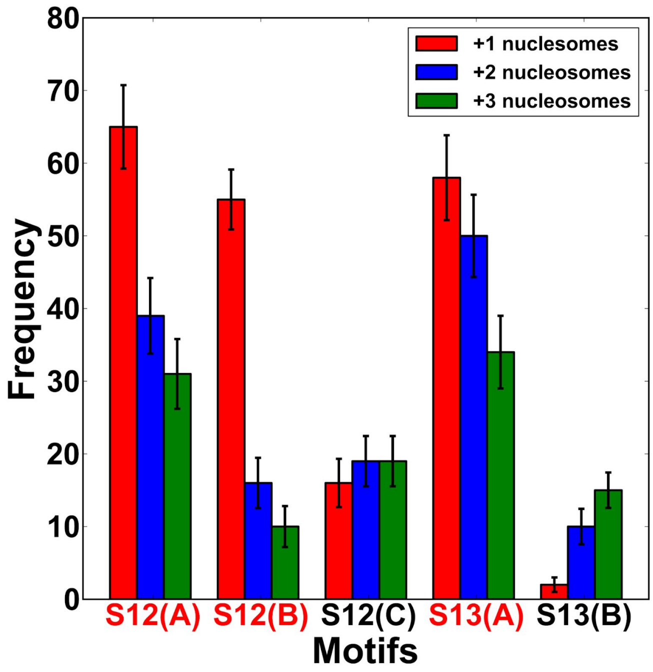

Supplementary Figure S18. Motif occupancy in +1 (red), +2 (blue), and +3 (green) nucleosomes for all 5 significant motifs (see asterisks in Supplementary Figures S12 and S13). A and/or T rich motifs (S12(A), S12(B), and S13(A)) are shown in red. P-values for a binomial test of +1 nucleosome motif occupancy (null hypothesis of 1/3 of motifs occuring in the +1 nucleosome) for each motif are 0.00047, 2.8×10^{-10}, 0.66, 0.062, and 0.0033, respectively.
